# Summarizing current refractory disease definitions in rheumatoid arthritis and polyarticular juvenile idiopathic arthritis: systematic review

**DOI:** 10.1093/rheumatology/keab237

**Published:** 2021-03-12

**Authors:** Hema Chaplin, Lewis Carpenter, Anni Raz, Elena Nikiphorou, Heidi Lempp, Sam Norton

**Affiliations:** 1 Health Psychology Section, Institute of Psychiatry, Psychology and Neuroscience, London, UK; 2 Centre for Rheumatic Diseases, King’s College London, London, UK

**Keywords:** refractory disease, non-response, treatment-resistant, rheumatoid arthritis, juvenile idiopathic arthritis, definitions

## Abstract

**Objectives:**

To identify how refractory disease (or relevant terminology variations) in RA and polyarticular JIA (polyJIA) is defined and establish the key components of such definitions.

**Methods:**

Searches were undertaken of English-language articles within six medical databases, including manual searching, from January 1998 to March 2020 (PROSPERO: CRD42019127142). Articles were included if they incorporated a definition of refractory disease, or non-response, in RA/polyJIA, with clear components to the description. Qualitative content analysis was undertaken to describe refractory disease in RA/polyJIA and classify each component within each definition.

**Results:**

Of 6251 studies screened, 646 met the inclusion criteria; 581 of these applied non-response criteria while 65 provided refractory disease definitions/descriptions. From the non-response studies, 39 different components included various disease activity measures, emphasizing persistent disease activity and symptoms, despite treatment with one or more biologic DMARD (bDMARD). From papers with clear definitions for refractory disease, 41 components were identified and categorized into three key themes: resistance to multiple drugs with different mechanisms of action, typically two or more bDMARDs; persistence of symptoms and disease activity; and other contributing factors. The most common term used was ‘refractory’ (80%), while only 16.9% reported explicitly how their definition was generated (e.g. clinical experience or statistical methods).

**Conclusion:**

Refractory disease is defined as resistance to multiple drugs with different mechanisms of action by persistence of physical symptoms and high disease activity, including contributing factors. A clear unifying definition needs implementing, as the plethora of different definitions makes study comparisons and appropriate identification of patients difficult.

**Rheumatology key messages**

- Refractory disease is multi-DMARD resistant with persistent symptoms and disease activity, including contributing factors.
- There is a lack of consensus in refractory disease definitions, with great heterogeneity.
- A unifying definition should be implemented, as a plethora of different definitions makes comparisons difficult.

## Introduction

### Rationale

The current ‘treat-to-(low disease activity)-target’ approach to care [1, 2] is successful in reducing inflammatory markers with DMARDs in up to two-thirds of patients, including people with severe, uncontrolled RA [3]. However, the impact on improving quality of life is considerably lower [4] and even those with low disease activity continue to experience persistent pain (40%) and fatigue (62%) [5, 6], with complex interactions between physical and mental health comorbidities and other contextual factors playing a role. Those who do not attain this low disease activity target are defined as having refractory disease [7, 8]. Another definition [non-response to three or more biologic DMARDs (bDMARDs)] identified 6% of a cohort as bDMARD refractory with a median time to the third bDMARD class of 8 years from starting the first anti-TNF [9].

The Collins English dictionary defines *refractory* as ‘unmanageable, stubborn or not responding/yielding to treatment in a medical context’ [10]. The use of various definitions or labels in both clinical practice and in the literature to describe these patient groups is problematic. For example, ‘treatment/therapy resistant’ [8], ‘difficult to treat’ [11, 12], ‘difficult to control’ [13], ‘fibromyalgic RA’ [14] and ‘treatment failure’ [15] have all been utilized in addition to ‘refractory’ [16]. The absence of a clear, routinely used definition or formal guidelines for refractory RA, especially juvenile onset, leaves patients and clinicians in a treatment vacuum [11, 17], without optimal bDMARD sequencing beyond a second bDMARD [9].

Previous definitions relate to MTX or conventional synthetic DMARDs (csDMARDs) [18], which may no longer be appropriate since further bDMARDs and targeted synthetic DMARDs (tsDMARDs) are now used in the management of both adult- and juvenile-onset inflammatory arthritis such as RA and polyarticular JIA (polyJIA). Moreover, there appears to be a lack of consistency between definitions, with one study identifying as few as 10% or as many as 28.8% of their population as refractory depending on which definition was used [19]. The absence of a systematic approach to identify, understand and evaluate refractory disease means that the true impact and underlying mechanisms remain unknown [16]. It is therefore timely to conduct a systematic review to identify the published components of definitions for refractory disease in RA/polyJIA and to evaluate these constituents for consistency in terminology in the future.

### Objectives

The objectives of this review were to assess how refractory disease (or relevant terminology variations) in RA/polyJIA is defined, classified and characterized in the literature and identify the key components of such definitions and group these constructs thematically.

## Methods

Cochrane [20] and Preferred Reporting Items for Systematic Reviews and Meta-Analyses (PRISMA) [21] guidelines were followed and data reported accordingly.

### Search strategy

Searches were undertaken of English-language articles within the Ovid (MEDLINE, Embase and PsycINFO), Cumulative Index to Nursing and Allied Health Literature (CINAHL), Web of Science and OpenGrey databases as well as manual searching of reference lists of included studies. As a sensitivity check, websites of relevant organizations were screened for additional definitions (e.g. ACR, American Registry of Medical Assistants, British Society for Rheumatology, National Institute for Health and Care Excellence and National Health Service England. Only articles published between January 1998 and March 2020 were included, due to the introduction of biologic treatments in 1998 [22–25], making research conducted before this time less comparable to current experiences of refractory disease.

Separate searches were carried out in each database (see Supplementary 1, available at *Rheumatology* online for further details). All search results (titles and abstracts) were exported into EndNote X8 software (Clarivate, London, UK) to be stored during the screening process. The search was conducted on 4 March 2020 and a study protocol was registered on the PROSPERO website (CRD42019127142; https://www.crd.york.ac.uk/prospero/display_record.php?ID=CRD42019127142).

### Eligibility criteria

Articles were included if they incorporated a definition of refractory disease (or any variants of these, e.g. treatment resistance or non-response) in RA/polyJIA, with clear components to the description. Articles with disease activity non-response criterion, such as ACR and EULAR, were included to capture components used for non-response, but were analysed separately as the main focus of the review was those articles with a more detailed definition for refractory disease. There were no restrictions on the types of studies to be included in the review, as long as a definition was operationalized. A full list of inclusion and exclusion criteria is shown in Table 1 using the Population, Intervention, Comparison, Outcome(s) and Study design framework [21].

**Table 1:** Eligibility criteria for considering studies for this review

	Inclusion criteria	Exclusion criteria
Population	RA PolyJIA Biologic drugs (e.g. etanercept/Enbrel, infliximab, Humira, anakinra, rituximab, tocilizumab etc.) or targeted synthetic therapies (e.g. Janus kinase inhibitors such as baricitinib or tofacitinib) explicitly stated as treatment	Other health conditions besides RA or polyJIA Acute health conditions or symptoms Non-inflammatory rheumatic disease (e.g. OA) Other inflammatory rheumatic disease (e.g. PsA, AS and uveitis) Treated with conventional synthetic drugs only (e.g. MTX alone)
Intervention/exposure (construct)	Refractory disease and any variations for this (e.g. treatment/therapy resistant, difficult/hard to control, non-responsive/response)	Disease that is being adequately controlled by treatment Acute symptoms that are adequately controlled by treatment
Comparison	Not applicable—studies with or without comparison groups included	Not applicable—studies with or without comparison groups included
Outcomes	Operationalized definition with clear components (either in the introduction, outcome variables, results or discussion)	No definition stated
Study design	Any study design (e.g. observational, interventional, qualitative studies, commentaries or reviews, policy documents)	Laboratory studies using animal models or cells

### Data collection and analysis

#### Selection of studies

A random sample of 10% of studies were cross-checked by a second coder (A.R.) at the screening stage, which resulted in a 0.77 level of agreement between the two coders [26]. Raters discussed discrepancies, revisited the criteria for inclusion that were outlined a priori and reached agreement on the final included studies for the review.

#### Data extraction and analysis

With the use of a study-specific data extraction table, information about each study (e.g. author, year of publication, country, study design/document type), patient population (e.g. disease name, adult/paediatric), definition details (e.g. title of definition, verbatim definition) and disease activity criteria were inserted. Study demographics and disease activity criteria were summarized and reported descriptively as counts and percentages, with figures created in Stata (version 16.1; StataCorp, College Station, TX, USA).
The verbatim definitions and identified non-response criteria were thematically coded for content, themes and patterns using content analysis to identify trends in definition components used and to quantify these by presenting frequencies of coded categories [27, 28]. The components within each definition (e.g. time specified, physician assessment, number of drugs required to classify non-response) were coded thematically, then compared and grouped until no new categories emerged [29, 30], using NVivo (version 12.6; QSR International, Chadstone, VIC, Australia). A second coder (H.L.) cross-checked initial coding and themes for consistency and reliability. A narrative synthesis of this qualitative content analysis is presented to describe refractory disease [31, 32]. Content overlap between studies was estimated using the Jaccard Index, which is a similarity coefficient for binary data that ranges from 0 (no overlap) to 1 (complete overlap) [33]. A network plot of co-occurrence of the most frequently used components and comparisons was generated using Stata (version 16.1).

#### Quality assessment of included studies

The quality of the included studies was assessed using the Hawker checklist [34], which is designed to appraise and score methodological quality [35] in disparate data from different methodologies. This was modified slightly for conference abstracts, which scored 1 for abstract/title and then other domains were assessed the same as if the conference abstract was a full-text article. A more rigorous risk of bias is not required, as this is a review aiming to determine how refractory disease is defined, classified and characterized in the literature [36], therefore studies of low quality are still included.

## Results

### Study selection

**Fig. 1:**
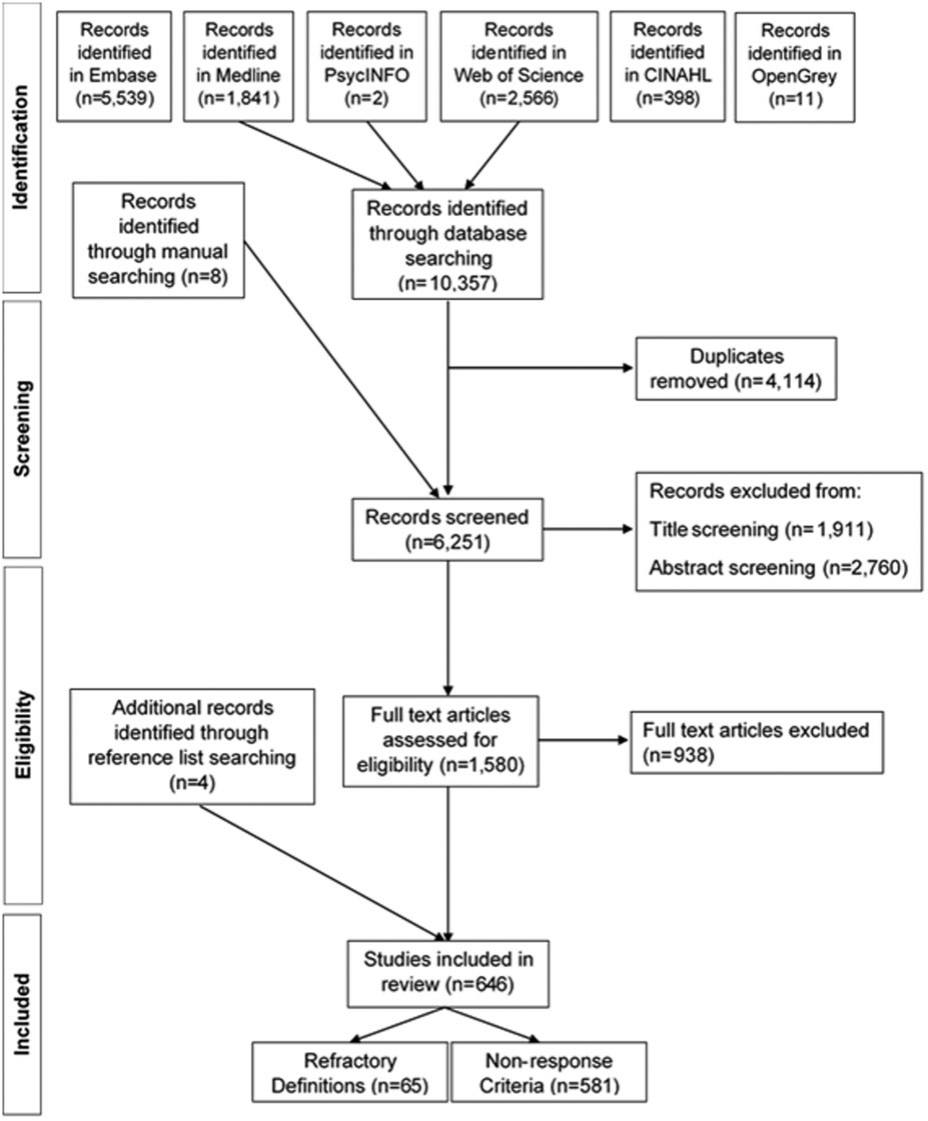
PRISMA flowchart of study selection

Combined searches yielded a total of 10 357 citations, of which 6251 remained after removal of duplicates (Fig. 1). Most citations were excluded due to not investigating either RA or polyJIA (*n* = 1600) or refractory disease or non-response (*n* = 2085). Full reasons for exclusion at each stage are presented in Supplementary Table 2, available at *Rheumatology* online. This left 646 studies meeting the inclusion criteria for this review, of which 581 reported non-response criteria and 65 reported refractory disease definitions/descriptions.

### Study characteristics

The majority of included studies (*n* = 646) investigated RA (91.5%) and adult (92.4%) populations, mainly from Europe (52.9%), utilizing a prospective observational design (33.3%) and published since 2010 (81.7%). For the non-response criteria papers (*n* = 581; see Supplementary Table 3, available at *Rheumatology* online), the majority investigated RA (92.8%) and adult (93.5%) populations, mainly from Europe (51.3%), utilizing a prospective observational design (35.8%) with a stable publication rate since 2006. The refractory definition papers (*n* = 65; see Supplementary Table 4, available at *Rheumatology* online) included more paediatric (16.9%) and polyJIA (20%) populations than the non-response, with a greater majority from Europe (67.7%), and in particular the UK (29.2%) and employed a case study/series design (20%) and publications have been increasing since 2006.

### Results of individual studies

#### Non-response criteria

The most frequently used disease activity measures to operationalize definitions for non-response to b/tsDMARDs for RA were the EULAR [37] (40%), DAS [38] [including the 28-joint (DAS28), 44-joint (DAS44) and juvenile arthritis (JADAS) [39]; 35%], 20% and 50% improvement in ACR criteria (ACR20 and ACR50; 16%) [40] and joint count (12%) non-response criteria. While for JIA these differed by using JIA-specific disease activity measures such as 30, 50 and 70% improvement in ACR Pediatric criteria (ACRPedi30, 50 and 70; 42.9%) [41], Wallace for non-remission (11.9%) [42] and JADAS (9.5%), uveitis consistently used the Standardization of Uveitis Nomenclature (SUN) criteria (19%) [43]. This is not unexpected, as non-response for specific disease criteria is not a requirement for treatment provision in JIA.

Overall, 39 different components were used in these non-response definitions/descriptions (see Supplementary 5, available at *Rheumatology* online), with various disease activity measures (85.6%). The majority applied a single criterion for disease activity (74.2%); the most popular were EULAR (38.5%), DAS28 (24.8%) and joint count (5.4%). Studies with two criteria (18.6%) typically cut-offs for DAS28 and/or EULAR (e.g. DAS28>2.6 or EULAR criteria for poor responders) (33.3%), joint count (6.5%) and/or ACR20 (5.6%), with 7.2% using more than two criteria to define non-response. Few included patient-reported outcome measures (5%), with a great variety and no clear preferences. Many studies used established cut-offs, with a minority that provided different values, particularly for the DAS (*n* = 7). The main descriptions/definitions of non-response could be summarized in two themes as emphasizing persistent disease activity and symptoms (93%), despite treatment with at least one bDMARD (typically a first-line anti-TNF) (Table 2).

**Table 2:** Subthemes and themes across definitions of non-response and refractory disease

Key themes		
Persistency of symptoms and disease activity^a^	Resistance to multiple drugs with different mechanisms of action^a^	Other contributing factors^b^
Subthemes		
Disease activity criteria^a^	Drug duration specified^a^	Other contributing factors^b^
Remission criteria^a^	Drugs/regimes failed, intolerant, discontinued or switched^a^	Biomechanical or degenerative drivers^b^
Patient-reported outcomes/symptoms^a^		Adverse event^b^
Presence or absence of inflammation^b^	Steroid use or dependency^a^	Comorbidities or extra-articular manifestations^b^
Disease severity^a^	Resistance to multiple drugs (regimes) with different structures or mechanisms of action^b^	Serology or antibodies^b^
New joint activity, damage or replacement^b^		Incorrect diagnosis or not relevant treatment^b^
Persistency of symptoms and disease activity^b^		

a Both non-response criteria and refractory definitions/descriptions.

b Refractory definitions/descriptions only.

#### Refractory definitions/descriptions

**Fig. 2:**
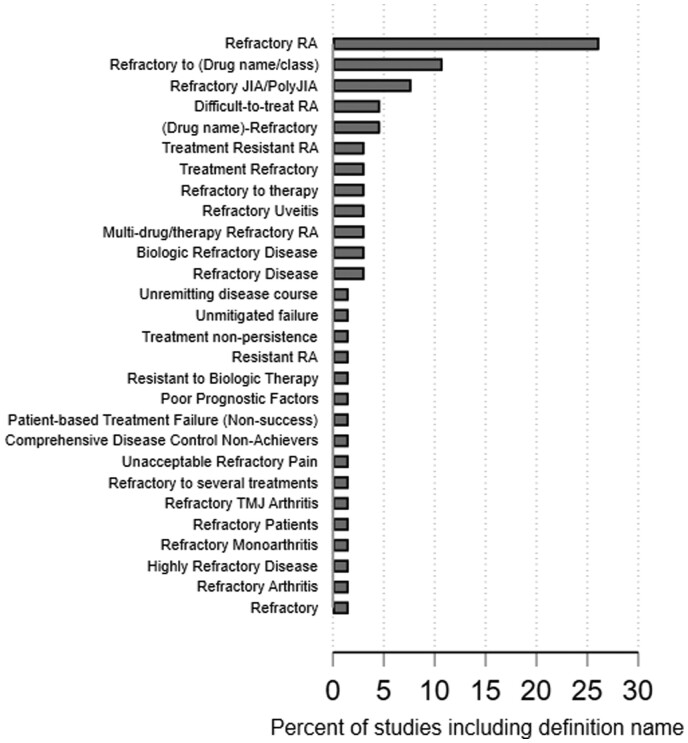
Chart representing the different terms/labels used (frequencies) across definitions

The characteristics of the 65 individual studies that reported a refractory disease definition or description [7, 9, 11, 12, 16, 44–103], including the verbatim definitions/descriptions, are presented in Supplementary Table 7, available at *Rheumatology* online. There was great variety in the definition name/labels used, but most incorporated the term ‘refractory’ (80%), while 20% used a variety of other terms, as seen in Fig. 2. Only 16.9% of included papers stated how their definition was generated; the majority (83.1%) did not provide any details. The 11 explanations of definition creation included clinical opinion/experience of the study authors (27.3%) [12, 16, 73], statistical analysis/modelling (18.2%) [95, 103], interdisciplinary panel discussion external to study authors (18.2%) [91, 94], rheumatology initiatives (18.2%) [7, 92], survey among rheumatologists (9.1%) [11] and from their previous work (9.1%) [60].

**Fig. 3:**
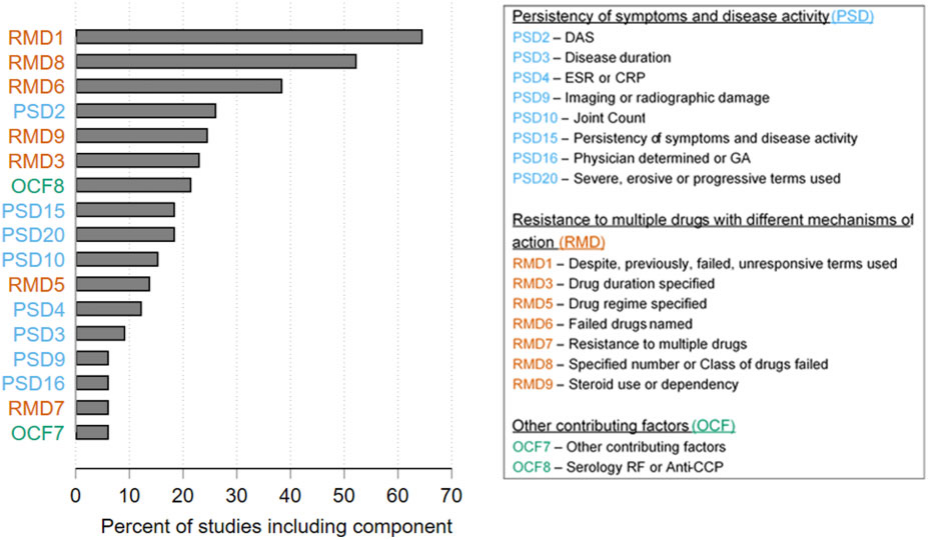
Most frequently occurring components across studies (17/41), with key for full component descriptions

**Fig. 4:**
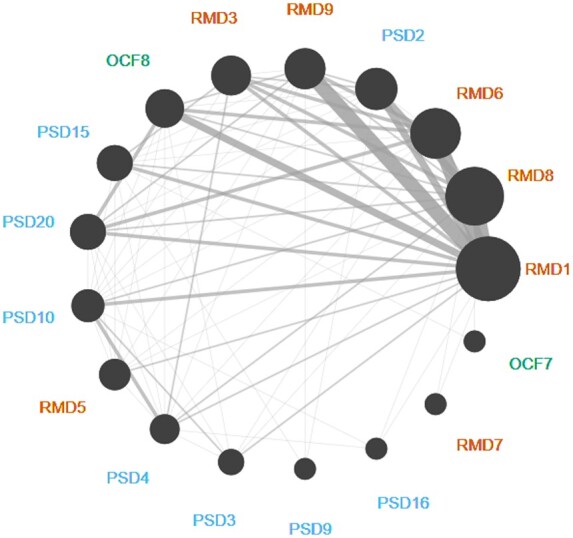
Network plot to demonstrate the frequency and occurrence of most frequently used components The size of each node is relative to the number of studies including the component, while the width of the connecting lines is relative to the number of co-occurrences. Components with less than three occurrences are excluded from the graph and lines omitted where co-occurrences were one. Key for components is in Fig. 3.

A total of 41 components were identified within these definitions (see Supplementary Tables 5 and 6, available at *Rheumatology* online); most list, on average, 4 distinct components (IQR 3–5; range 1–10) per definition. The dimensions were categorized, coded thematically and quantified and are displayed in Table 2 within three key themes: resistance to multiple drugs with different mechanisms of action (54.9%), persistency of symptoms and disease activity (34.9%) and other contributing factors (10.2%). There was great variation in the components used across definitions, with no clear consistent patterns aside from the majority of studies incorporating multi-bDMARD resistance, as seen in Figs 3 and 4.

Overlap among all 41 components both within and across studies was estimated via the Jaccard Index correlation coefficient. There was a very weak similarity (0.19) in the overlap of components within studies, while there was nearly no similarity (0.05) in the clustering of components across studies, making comparisons of the level of agreement between patients satisfying different definitions impossible. However, as seen in Fig. 4, there seems to be several key components that are frequently used together across studies, which all relate to resistance to multiple drugs (theme 2), including steroids, and persistent disease as determined by the DAS.

For RA, most definitions contained three to four components, of which the most commonly used were ‘Despite, previously, failed, unresponsive terms used’ (RMD1, 16.4%) to indicate treatment resistance, ‘Specified number or Class of drugs failed’ (RMD8, 13.6%) and ‘Failed drugs named’ (RMD6, 8.2%) (Fig. 3). For polyJIA, most definitions contained four components and these differed slightly, as the most commonly used were ‘Steroid use or dependency’ (RMD9, 13%), which may reflect the reliance of steroids with more limited treatment guidelines in JIA than RA, jointly with ‘Failed drugs named’ (RMD6, 13%) and ‘Despite, previously, failed, unresponsive terms used’ (RMD1, 11.1%).

Two studies discussed refractory symptoms in the presence of controlled inflammation; Olofsson *et al.* [60], which described ‘unacceptable refractory pain’, and Buch [16], which described ‘false refractory disease compared with biologically refractory disease either intrinsic or pharmacokinetic’. Most studies defined refractory disease as affecting multiple joints, ranging from 4 to 24 joints [67, 98], with most requiring 6 or 8 joints [45, 49, 78, 79, 85, 94] or involvement of the large joints specifically [47]. Two studies reported only one affected joint [54, 87]. Wolfe *et al.* [103] used a patient outcome–based definition, although without explanation as to how patients were involved in selecting these outcomes; overall patient-reported outcomes represented only 3.2% of the components used.

For those definitions that named specific drugs that had been failed, the number of drugs ranged from one to eight, although two were mentioned on average. The majority of these were bDMARDs, usually anti-TNF more than the other classes, followed by csDMARDs and bDMARDs and steroids with either bDMARDs or both bDMARDs and tsDMARDs. For those definitions that specified the number of drugs that failed, the number of these ranged from one to six, with three mentioned on average. The majority of these were bDMARDs, followed by csDMARDs and bDMARDs, anti-TNF bDMARDs and not specifying which class/type beyond stating immunosuppression.

The few earlier studies conducted between 1999 and 2005 (*n* = 6) incorporated more components per definition [median 5.5–7.5 (range 3–10)] than the studies conducted since then (*n* = 59; 2006 to date), which report 3–4 components on average (range 1–8), although this difference did not reach significance [*F*(5,59) = 2.14, *P* = 0.07]. Because of this, there has been a slight shift in the frequency per type of components reported in definitions over time [*F*(3,61) = 2.28, *P* = 0.08, *R*^2^ = 0.1], with the number of components relating to persistency of symptoms and disease activity (theme 2) decreasing per definition [*t* = −2.59 (95% CI −0.55, −0.07); *P* = 0.012], while the other two themes have remained stable.

In particular, reporting of the following components has decreased over time (*P*<0.05): DAS; functional score; joint damage or replacement; patient global, severe, erosive or progressive terms; stiffness; and serology RF or anti-CCP. However, there were no notable differences between different countries for either the total number of components used in reported definitions [*F*(6,58) = 1.09, *P* = 0.38] or types of components reported as grouped by the three themes [*F*(3,61) = 0.20, *P* = 0.89, *R*^2^ = 0.01]. Some differences were found in that only definitions from Asia (*n* = 6) and the Middle East (*n* = 1) mentioned switching drugs (*P* = 0.012), while only definitions from Europe, the UK and North America incorporated serology RF or anti-CCP (*P* = 0.066).

### Quality assessment of included studies

The quality of the 65 studies that included a refractory definition/description was assessed using the Hawker checklist (see Supplementary Table 8, available at *Rheumatology* online), which found that 10 articles were of high quality and 13 were of medium quality, but the majority were considered low quality (*n* = 42). The areas in which the articles performed the best were in relation to results, methods and data, and implications and usefulness, while the worst areas were ethics and bias, data analysis and sampling.

## Discussion

### Summary of evidence

The aim for this review was to identify how definitions of refractory disease in RA/polyJIA are operationalized and the key components included in these definitions. During the search it became clear that non-response to b/tsDMARDs can be operationalized using disease activity response criteria or more detailed descriptions/definitions labelled as refractory disease. A wide range of criteria defined non-response, and despite a lack of consistency, the most widely used were EULAR, DAS28 and joint count. It seems that EULAR was the most popular, as the majority were conducted in Europe. Attention was paid to other patient-reported outcomes such as pain, functional assessments and fatigue, but these were minimal.

From the 581 non-response studies, 39 different components were identified that included various disease activity measures, e.g. persistent disease activity and symptoms, despite treatment typically with at least one bDMARD (typically anti-TNF). From the 65 articles detailing definitions for refractory disease, 41 components were identified and broadly categorized into three key themes: resistance to multiple drugs with different mechanisms of action, typically at least two bDMARDs; persistency of symptoms and disease activity; and other contributing factors. Refractory disease is not consistently defined; instead, a broad range and variations of criteria or components are arbitrarily chosen, with generation of these definitions mostly not specified. The current definitions are medically focused, with fewer components over time, with little to no inclusion of psychosocial components, aside from pain and fatigue.

The majority of articles investigated RA and only a small proportion investigated refractory disease in polyJIA. This suggests that although refractory disease is prevalent in this population, it is currently underresearched. There has been growing interest since 2006 to investigate refractory disease in these two inflammatory arthritis conditions and attempts to define the construct. In contrast, publications about non-response using disease activity criteria have remained steady since 2006. This signifies that the concept of refractory disease goes beyond non-response as determined by disease activity measures/criteria.

With regards to the terms used to describe this patient group, it became clear that ‘refractory’ is the most popular term in the rheumatology literature. However, more work is needed to investigate whether ‘refractory’ is a patient-friendly term that is easily understandable and acceptable to describe their difficult-to-treat inflammatory disease. Patients tend to define and rate their illness differently from medical professionals [104], which in turn influences their opinions of treatment efficacy [105] and achieving agreed treatment targets, including their perception of remission [106, 107]. Thus patients’ understanding of refractory disease needs to be explored to incorporate their experiences and perceptions to consider their unmet needs, both through research participation and involvement in study priorities and design [108, 109].

The credibility and validity of the descriptions presented here are questionable, as the majority of authors did not state how they had generated their definition. Although two citations used a more stringent and independent method of definition generation through interdisciplinary panels for refractory disease in polyJIA and polyJIA–uveitis [91, 94], more details about the exact panel process and involved disciplines were needed. Beukelman *et al.* [94] used a formalized process in their guidelines development and involved a nurse, a general paediatrician and a parent, for example, to represent non-rheumatologists. It remains unclear how many rheumatologists were involved. Bou *et al.* [91] failed to provide any details about professional roles of their panel or exact details of the panel process.

All patients with RA or polyJIA require the support of a multidisciplinary team in addition to their rheumatologist, particularly those with refractory disease [9, 12], yet only one of these definitions was developed with the involvement of non-rheumatology healthcare professionals [94]. Future research may employ other methods of definition generation, such as the Delphi consensus voting method, which allows a range of experts from different disciplines to provide insights and expertise and is routinely used in rheumatology for the generation of outcome measures [110, 111], classification criteria [112] and reporting guidelines [113].

The quality of reporting of refractory disease, and in particular papers that propose operational definitions/descriptions, needs to be improved, as evidenced by the majority of studies identified as low quality using the Hawker checklist. This may be due in part to the nature of the study designs included in this review, as conference abstracts (*n* = 17) have limited word counts and reporting such details is not the focus of case studies (*n* = 13), which were more prevalent in this review. Papers that scored as high quality were often randomized controlled trials (*n* = 6) with more detailed reporting. Nonetheless, future studies need to include all aspects of study reporting highlighted by the Hawker checklist to determine quality and allow replication and validation.

### Limitations

There are a number of limitations with this review. Although discrepancies were discussed before reaching final agreement, the percentage of agreement between raters was moderate. This highlights that refractory disease is a complex construct to understand, evaluate and define. Although the Hawker checklist was the most appropriate tool for the aim of this review, the score did not fully assess the quality of all studies such as conference abstracts or case studies, where the details required for assessment are typically limited. Perhaps another tool needs to be developed to properly assess quality in a range of different article types, including conference abstracts, for such reviews to take into account disparate data across a range of literature types.

Finally, this review focused on refractory disease in people with RA and polyJIA. Refractory disease is present in many physical and mental health long-term conditions [114–117], including inflammatory arthritis and related rheumatic conditions [118, 119]. A future review could expand to encompass all refractory inflammatory arthritis conditions, with inclusion of other paediatric-onset conditions. This approach would allow comparisons and the identification of common constructs across a wide range of conditions.

## Conclusions and implications

Refractory disease can be defined as resistance to multiple drugs with different mechanisms of action as evidenced by persistency of symptoms and disease activity with other contributing factors. Current definitions have appropriately focused on biological processes. In conjunction with this approach, wider psychosocial components need to be incorporated [120, 121]. Some authors directly advocated for a broader definition highlighting the importance of wider factors such as comorbidities [13, 122]. A definition of refractory disease needs to include additional factors beyond non-response to a specified number of bDMARDs to truly reflect this group of patients. This would allow the definition to be universal and not constrained by country-specific restrictions on access to treatments while also remaining flexible to anticipate increasing treatment options and availability [13].

The growing number of publications about refractory disease in rheumatology, and most recently the EULAR Task Force on Difficult-to-Treat RA [123], highlights the need to further identify, consolidate and implement additional components of refractory disease through consensus methods and/or conferences. This would enable a detailed understanding about this group of patients, their treatment expectations and experiences of non-response against the background of the increasing number of treatment options and the prospect of personalized medicine. This review has highlighted current definitions identified as important to characterize refractory disease but also recognizes further areas to be investigated.
The plethora of different definitions makes both study comparisons and appropriate identification of patients difficult. A clear implementable definition for refractory disease is important for rheumatologists and commissioners to be able to design and commission appropriate services and allocate resources for patients affected by the condition. A way forward could be the routine establishment of multidisciplinary refractory clinics to allow in-depth discussion and exploration of treatment options, beyond standard care (if recommended treatments/regimes have already been tried) with an holistic non-pharmacological focus rather than simply increasing/adding drugs, which may not be appropriate.

## Supplementary Material

keab237_Supplementary_Data
