# Supplementary material for: Summarizing current refractory disease definitions in rheumatoid arthritis and polyarticular juvenile idiopathic arthritis: systematic review
Source: Rheumatology (Oxford). 2021 Mar 12;60(8):3540–52. doi: 10.1093/rheumatology/keab237 (PMC8328502; doi:10.1093/rheumatology/keab237)
Supplement: keab237_Supplementary_Data [file keab237_supplementary_data.zip › rhe-20-2738-File002.docx]

Supplementary Data S1 – Search Strategy

| Refractory terms | Health Conditions | Drug |
| --- | --- | --- |
| Refractory  Treatment Resistan*  Therapy Resistan*  Drug Resistan*  Biologic Resistan*  Treatment Non-respon*  Therapy Non-respon*  Non-respon*  Inadequate respon*  Poor respon*  Unrespon*  Treatment failure  Therapy failure  Difficult to treat  Hard to treat  Difficult to control  Hard to control  Non-remission  Poor*-control*  Unremit* | Rheumatoid Arthritis  Polyarticular Juvenile Idiopathic Arthritis  Polyarthritis  NOT  Osteoarthritis  Still* Disease  Sjogren*  Psoriatic Arthritis  Ankylosing Spondylitis  Spondylarthritis  Systemic Lupus Erythematosus/Lupus  Lyme  Septic Arthritis  Enteropathic Arthritis  Reactive Arthritis  Polymyalgia Rheumatica  Giant Cell Arteritis  Vasculitis  Scleroderma | Disease-Modifying Anti-Rheumatic Drug?  ??DMARD?  (Antirheumatic Agent MH)  Biologic Treatment  Biologic Therapy  Targeted Synthetic Treatment  Targeted Synthetic Therapy  Janus Kinase Inhibitor  JAK Inhibitor |

Medline, Embase and PsycInfo MEDLINE via Ovid search strategy

1 Refractory.ab,ti

2 Treatment Resistan*.ab,ti

3 Therapy Resistan*.ab,ti

4 Drug Resistan*.ab,ti

5 Biologic Resistan*.ab,ti

6 Treatment Non-respon*.ab,ti

7 Therapy Non-respon*.ab,ti

8 Non-respon*.ab,ti 9 Inadequate respon*.ab,ti

10 Poor respon*.ab,ti

11 Unrespon*.ab,ti

12 Treatment failure.ab,ti

13 Therapy failure.ab,ti

14 Difficult to treat.ab,ti

15 Hard to treat.ab,ti

16 Difficult to control.ab,ti

17 Hard to control.ab,ti

18 Non-remission.ab,ti

19 Poor*-control*.ab,ti

20 Unremit*.ab,ti

21 OR/1-20

22 exp Arthritis, Rheumatoid/

23 (Rheumatoid adj3 Arthritis).ab,ti

24 exp Juvenile Rheumatoid Arthritis/

25 (Juvenile adj3 Arthritis).ab,ti

26 Polyarticular Juvenile Idiopathic Arthritis.ab,ti

27 Polyarticular JIA.ab,ti

28 Polyarthritis.ab,ti

29 OR/22-28

30 exp Antirheumatic Agent/

31 Disease-modifying anti-rheumatic drug?.ab,ti.

32 ??DMARD?.ab,ti

33 Biologic Treatment.ab,ti

34 Biologic Therapy.ab,ti

35 Targeted Synthetic Treatment.ab,ti

36 Targeted Synthetic Therapy.ab,ti

37 Janus Kinase Inhibitor.ab,ti

38 JAK Inhibitor.ab,ti

39 OR/30-38

40 21 AND 29 AND 39

41 Limit 40 to English Language

42 Limit 41 to Human

43 Limit 42 to yr=”1998-Current”

60 remove duplicates from 59
