# Supplementary material for: Summarizing current refractory disease definitions in rheumatoid arthritis and polyarticular juvenile idiopathic arthritis: systematic review
Source: Rheumatology (Oxford). 2021 Mar 12;60(8):3540–52. doi: 10.1093/rheumatology/keab237 (PMC8328502; doi:10.1093/rheumatology/keab237)
Supplement: keab237_Supplementary_Data [file keab237_supplementary_data.zip › rhe-20-2738-File003.docx]

Supplementary Table S2 – Reasons for exclusion at screening

| Reasons for exclusion | Titles | Abstracts | Full Texts |
| --- | --- | --- | --- |
| Not RA/PolyJIA | 1430 | 147 | 23 |
| No b/tsDMARD | 222 | 128 | 15 |
| Not Refractory/Non-response | 135 | 1800 | 151 |
| No Definition | 0 | 425 | 476 |
| Not Human | 22 | 11 | 0 |
| Not Article/Report | 10 | 0 | 3 |
| Before 1998 | 19 | 1 | 2 |
| No full text | 0 | 15 | 50 |
| Not English | 2 | 9 | 26 |
| Duplicate | 0 | 0 | 79 |
| Multiple reasons | 67 | 224 | 112 |
