# Supplementary material for: Summarizing current refractory disease definitions in rheumatoid arthritis and polyarticular juvenile idiopathic arthritis: systematic review
Source: Rheumatology (Oxford). 2021 Mar 12;60(8):3540–52. doi: 10.1093/rheumatology/keab237 (PMC8328502; doi:10.1093/rheumatology/keab237)
Supplement: keab237_Supplementary_Data [file keab237_supplementary_data.zip › rhe-20-2738-File004.docx]

Supplementary Table S3 – Study Characteristics of non-response criteria (n=581)

| Study Characteristics | Frequency (%) |
| --- | --- |
| Year of Publication  1998-2001  2002-2005  2006-2009  2010-2013  2014-2017  2018-date (March 2020) | 3 (0.52%)  23 (3.96%)  79 (13.60%)  162 (27.88%)  210 (36.14%)  104 (17.90%) |
| Disease Studied  RA  RA and PolyJIA  PolyJIA  PolyJIA-Uveitis | 539 (92.77%)  31 (5.34%)  1 (0.17%)  10 (1.72%) |
| Population  Adult  Paediatric  Adult PolyJIA | 597 (92.41%)  49 (7.59%)  5 (0.77%) |
| Country  Africa  Asia  Australia and New Zealand  Europe  Middle East  Not clear  North America  South America  Russia  Worldwide | 4 (0.69%)  113 (19.45%)  3 (0.52%)  298 (51.29%)  4 (0.69%)  5 (0.86%)  53 (9.12%)  5 (0.86%)  8 (1.38%)  84 (14.46%) |
| Study Design/Publication  Prospective Observational Study  Conference Abstracts  Randomised Controlled Trial  Retrospective Cohort Analysis  Secondary Trial Analysis  Open Label Study  Prospective Cohort Study  Other Retrospective Analysis  Genetic Study  Biomarker Analysis  Review (Literature or Systematic)  Analysis of Claims Database  Treatment Recommendations/Guidelines  Retrospective Observational Study  Cost-effectiveness Study  Case Study/Series  Economic/Decision Modelling  Viewpoint/Editorial  Cohort Study (Prospective and Retrospective)  Metabolomic Profiling | 208 (35.80%)  179 (30.81%)  86 (14.80%)  55 (9.47%)  48 (8.26%)  42 (7.23%)  33 (5.68%)  26 (4.48%)  17 (2.93%)  10 (1.72%)  9 (1.55%)  8 (1.38%)  7 (1.20%)  6 (1.03%)  4 (0.69%)  4 (0.69%)  2 (0.34%)  2 (0.34%)  1 (0.17%)  1 (0.17%) |
| Criteria Type  Non-remission  Non-response  Non-response (primary and secondary failure)  Non-remission and Non-response  Non-response and Treatment Failure  Treatment Criteria  Treatment Criteria and Non-remission  Treatment Criteria and Non-response  Treatment Failure | 28 (4.82%)  418 (71.94%)  23 (3.96%)  3 (0.52%)  3 (0.52%)  68 (11.70%)  1 (0.17%)  22 (3.79%)  15 (2.58%) |
| Response Criteria Used  Single Criteria Used  ACR  ACR20  ACR50  ACRPedi  ACRPedi30  ACRPedi50  ACRPedi70  CDAI  Curtis Claim  DAS28  DAS28-CRP  DAS28-ESR  DAS44  Drugs named/specified  Drug regimen specified  EULAR  HAQ-DI  JADAS  JADAS-10  Joint Count  Number of drugs specified  Patient Global  Physician Determined  Power Doppler  SDAI  Serum Interferon Ratio  Side Effects  Steroid Use  SUN  Treatment Switch/Discontinuation  Wallace  WPAI  Two Criteria Used  ACR20 and ACR50  ACR20 and CRP  ACR20 and DAS28  ACR20 and EULAR  ACR20 and CDAI/SDAI  ACR50 and EULAR  ACRPedi30 and EULAR  ACRPedi50 and Wallace  Boolean and DAS28  CDAI/SDAI and DAS28  CDAI/SDAI and Joint Count  DAS28 and ACR  DAS28 and EULAR  DAS28 and Joint Count  DAS28 and Power Doppler  Drug alteration/discontinuation and steroid use  Drug regimen specified and Joint Count  Drugs specified and Joint Count  EULAR and ACR  EULAR and Blood Biomarkers  EULAR and Drug alteration/discontinuation  EULAR and Drugs specified  EULAR and RADAI  EULAR and RAPID3  EULAR and CDAI/SDAI  JADAS and Steroid Use  Joint Count and ACR50  Joint Count and CDAI/SDAI  Joint Count and ESR/CRP  Joint Count and PatientGlobal/Pain VAS  Number of drugs and Joint Count  Physician Global and EULAR  Presence of AC cells and Steroid Use  RAPID3 and Drug discontinuation  Time and DAS28  Vascular changes and EULAR  More than two criteria used | 431 (74.18%)  3 (0.70%)  31 (7.19%)  13 (3.02%)  3 (0.70%)  6 (1.39%)  4 (0.93%)  1 (0.23%)  12 (2.78%)  8 (1.86%)  107 (24.83%)  8 (1.86%)  7 (1.62%)  1 (0.23%)  3 (0.70%)  3 (0.70%)  166 (38.52%)  2 (0.46%)  1 (0.23%)  1 (0.23%)  23 (5.34%)  4 (0.93%)  2 (0.46%)  2 (0.46%)  2 (0.46%)  2 (0.46%)  1 (0.23%)  1 (0.23%)  2 (0.46%)  6 (1.39%)  2 (0.46%)  3 (0.70%)  1 (0.23%)  108 (18.59%)  2 (1.85%)  1 (0.93%)  6 (5.56%)  5 (4.63%)  1 (0.93%)  1 (0.93%)  1 (0.93%)  1 (0.93%)  2 (1.85%)  3 (2.78%)  5 (4.63%)  2 (1.85%)  36 (33.33%)  7 (6.48%)  1 (0.93%)  1 (0.93%)  2 (1.85%)  3 (2.78%)  1 (0.93%)  1 (0.93%)  4 (3.70%)  2 (1.85%)  1 (0.93%)  1 (0.93%)  1 (0.93%)  1 (0.93%)  1 (0.93%)  3 (2.78%)  5 (4.63%)  1 (0.93%)  1 (0.93%)  1 (0.93%)  1 (0.93%)  1 (0.93%)  1 (0.93%)  1 (0.93%)  42 (7.23%) |
