# Supplementary material for: Summarizing current refractory disease definitions in rheumatoid arthritis and polyarticular juvenile idiopathic arthritis: systematic review
Source: Rheumatology (Oxford). 2021 Mar 12;60(8):3540–52. doi: 10.1093/rheumatology/keab237 (PMC8328502; doi:10.1093/rheumatology/keab237)
Supplement: keab237_Supplementary_Data [file keab237_supplementary_data.zip › rhe-20-2738-File005.docx]

Supplementary Table S4 – Study Characteristics of Refractory Disease papers (n=65)

| Study Characteristics | Frequency (%) |
| --- | --- |
| Year of Publication  1998-2001  2002-2005  2006-2009  2010-2013  2014-2017  2018-date (March 2020) | 2 (3.08%)  4 (6.15%)  7 (10.77%)  13 (20%)  19 (29.69%)  20 (30.77%) |
| Disease Studied  RA  PolyJIA  PolyJIA-Uveitis | 52 (80.00%)  12 (18.46%)  1 (1.54%) |
| Population  Adult  Paediatric  Adult PolyJIA | 54 83.08%  11 16.92%  2 3.08% |
| Country  Asia  Europe  Middle East  North America  South America  Worldwide | 6 (9.23%)  44 (67.69%)  1 (1.54%)  8 (12.31%)  2 (3.08%)  3 (4.62%) |
| Study Design/Publication  Conference Abstracts  Case Study/Series  Retrospective Cohort Analysis  Open Label Study  Prospective Observational Study  Randomised Controlled Trial  Secondary Trial Analysis  Treatment Recommendations/Guidelines  Review (Literature or Systematic)  Analysis of Claims Database  Prospective Cohort Study  Retrospective Observational Study  Trial Protocol  Viewpoint/Editorial | 17 (26.15%)  13 (20.00%)  12 (18.46%)  10 (15.38%)  8 (12.31%)  6 (9.23%)  4 (6.15%)  4 (6.15%)  2 (3.08%)  1 (1.54%)  1 (1.54%)  1 (1.54%)  1 (1.54%)  1 (1.54%) |
