# Supplementary material for: Summarizing current refractory disease definitions in rheumatoid arthritis and polyarticular juvenile idiopathic arthritis: systematic review
Source: Rheumatology (Oxford). 2021 Mar 12;60(8):3540–52. doi: 10.1093/rheumatology/keab237 (PMC8328502; doi:10.1093/rheumatology/keab237)
Supplement: keab237_Supplementary_Data [file keab237_supplementary_data.zip › rhe-20-2738-File006.docx]

Supplementary Table S5: Components, Subthemes and Themes across Non-Response Criteria and Refractory Disease Definitions

| Non-Response Criteria | | | Refractory Disease Definitions | | |
| --- | --- | --- | --- | --- | --- |
| ACR | Disease Activity Criteria | Persistency of symptoms and disease activity  Persistency of symptoms and disease activity | ACR | Disease Activity Criteria | Persistency of symptoms and disease activity |
| CDAI/SDAI |  |  | DAS |  |  |
| CRP/ESR/Biomarkers |  |  | ESR CRP |  |  |
| DAS28/44 |  |  | Functional Score |  |  |
| EULAR |  |  | HAQ or CHAQ |  |  |
| JADAS |  |  | Joint Count |  |  |
| Joint Count |  |  | Physician determined or GA |  |  |
| Physician Determined |  |  | SDAI or CDAI |  |  |
| Serum Interferon Ratio |  |  | SUN |  |  |
| Power Doppler |  |  | Imaging or radiographic damage | PROs / Symptoms |  |
| SUN and ocular specific |  |  | Fatigue |  |  |
| Boolean | Remission Criteria |  | Pain |  |  |
| Wallace |  |  | Patient Global |  |  |
| Fatigue | Patient Reported Outcomes / Symptoms  Patient Reported Outcomes / Symptoms |  | RAPID3 |  |  |
| HAQ/HAQDI |  |  | Stiffness |  |  |
| Morning stiffness |  |  | Failure to achieve disease remission | Not achieving remission |  |
| Pain |  |  | Presence or absence of inflammation | Presence or absence of inflammation |  |
| Patient Activity Scale |  |  | Severe, erosive or progressive terms used | Disease severity |  |
| Patient Global |  |  | Disease duration |  |  |
| RADAI |  |  | Joint damage or replacement | New joint activity, damage or replacement |  |
| Radiographic damage/erosion |  |  | New joint activity |  |  |
| Range of movement |  |  | Persistency of symptoms and disease activity | Persistency of symptoms and disease activity |  |
| RAPID3 |  |  | Drug duration specified | Drug duration specified | Resistance to multiple drugs with different structures or mechanisms |
| SF-36 |  |  | Drug discontinuation | Drugs/regimes failed, intolerant, discontinued or switched |  |
| Swelling/Synovitis |  |  | Drug regime specified |  |  |
| WPAI |  |  | Drug intolerance or toxicity |  |  |
| Co-morbidities | Other Disease Severity Factors |  | Failed drugs named |  |  |
| Disease Duration |  |  | Switching drugs |  |  |
| Side Effects |  |  | Steroid use or dependency | Steroid use or dependency |  |
| Severe/active/erosive RA |  |  | Resistance to multiple drugs with different structures or mechanisms | Resistance to multiple drugs with different structures or mechanisms |  |
| Vascular changes |  |  | Specified number or Class of drugs failed |  |  |
| Drug Duration | Drug Duration | Resistance to multiple drugs (regimes) with different structures or mechanisms | Despite, previously, failed, unresponsive terms used |  |  |
| Curtis Claim | Drugs/regimes failed, intolerant, discontinued or switched |  | Other contributing factors | Other contributing factors | Other contributing factors |
| Drug intolerance |  |  | Biomechanical or degenerative drivers | Biomechanical or degenerative drivers |  |
| Drug regimen specified |  |  | Adverse event | Adverse event |  |
| Drugs named/specified |  |  | Co-morbidities or extra-articular manifestations | Co-morbidities or extra-articular manifestations |  |
| Number of drugs specified |  |  | Serology RF or Anti-CCP | Serology or anti-bodies |  |
| Treatment Switch/Discontinuation |  |  | Anti-drug Antibodies |  |  |
| Steroid Use/Dependency | Steroid Use/Dependency |  | Checking diagnosis | Incorrect diagnosis or not relevant treatment |  |
|  |  |  | Checking relevant treatment given |  |  |
|  |  |  | Treatment as outpatient |  |  |
