# Supplementary material for: Summarizing current refractory disease definitions in rheumatoid arthritis and polyarticular juvenile idiopathic arthritis: systematic review
Source: Rheumatology (Oxford). 2021 Mar 12;60(8):3540–52. doi: 10.1093/rheumatology/keab237 (PMC8328502; doi:10.1093/rheumatology/keab237)
Supplement: keab237_Supplementary_Data [file keab237_supplementary_data.zip › rhe-20-2738-File007.docx]

Supplementary Figure S6: Incidence of 41 components (columns) of Refractory Disease identified across 65 studies included in the review (rows)


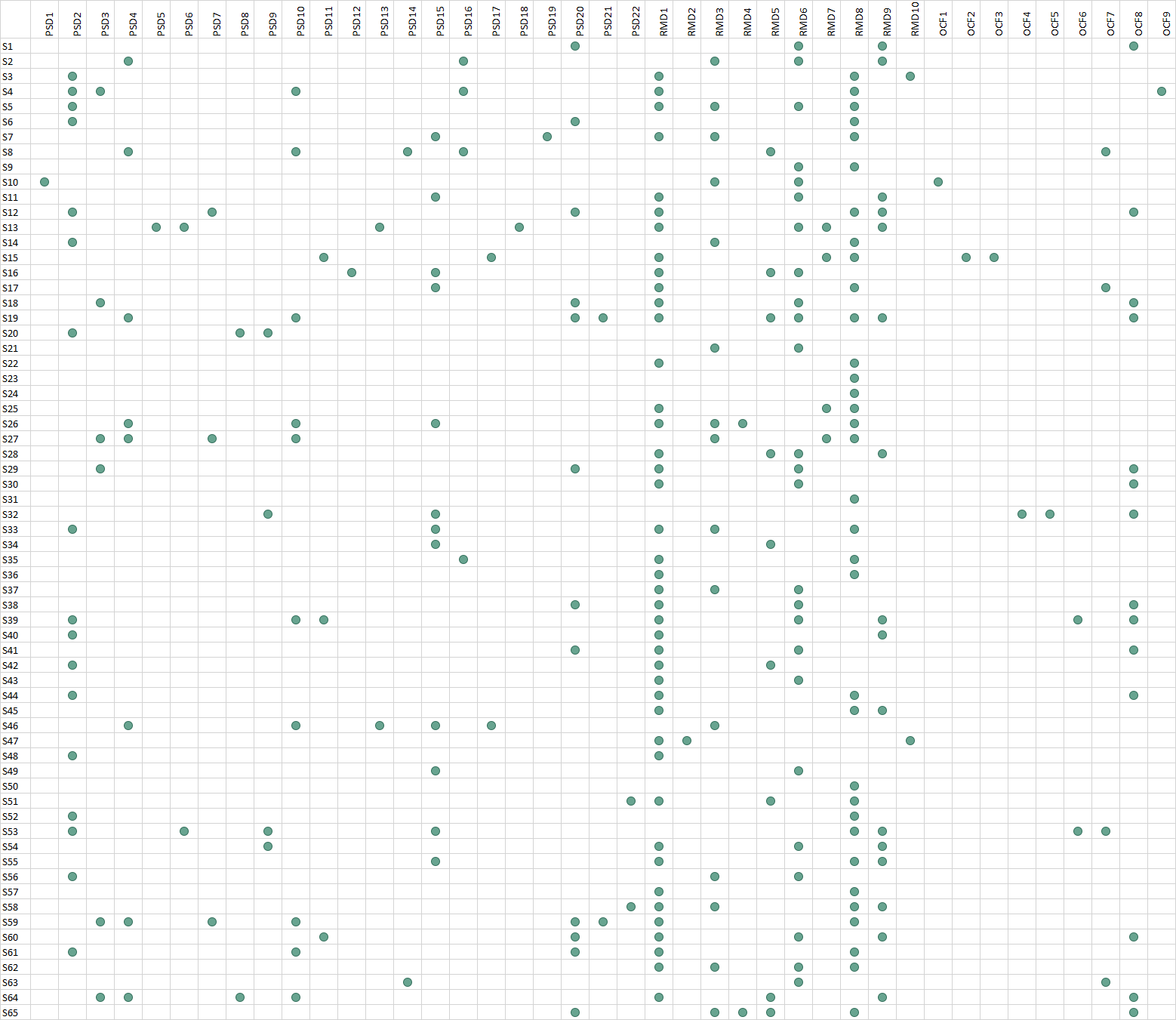


Table S6 – Key linking StudyID with Author Details and ComponentID with Component Details

| Study ID – Author (Ref) | ComponentID – Component Details |
| --- | --- |
| S1 – Abinun (101)  S2 – Albers (100)  S3 – Al Herz (99)  S4 – Alvaro-Gracia (98)  S5 – Arjun (97)  S6 – Baxter (96)  S7 – Bécède (95)  S8 – Beukelman (94)  S9 – Blazina (93)  S10 – Boers (92)  S11 – Bou (91)  S12 – Breban (90)  S13 – Brown (89)  S14 – Brulhart (88)  S15 – Buch (16)  S16 – Carubbi (87)  S17 – de Hair (12)  S18 – Di Poi (86)  S19 – Eklund (86)  S20 – Emery (102)  S21 – Farah (84)  S22 – Fernandes (83)  S23 – Fitton (82)  S24 – FitzGerald (81)  S25 – Gaylis (80)  S26 – Genovese (79)  S27 – Gillis (78)  S28 – Gomez (77)  S29 – Hashmi (76)  S30 – Hayes (75)  S31 – Heaf (74)  S32 – Isaacs (73)  S33 – Jois (72)  S34 – Katsicas (71)  S35 – Kawashiri (70)  S36 – Kearsley-Fleet (9)  S37 – Klimiuk (69)  S38 – Koumakis (68)  S39 – Kuek (67)  S40 – Liang (66)  S41 – Malaviya (65)  S42 – Marchesoni (64)  S43 – Marketos (63)  S44 – Moeller (62)  S45 – NHS England (61)  S46 – Olofsson (60)  S47 – Park (59)  S48 – Polido-Pereira (7)  S49 – Pontikaki (58)  S50 – Pope (57)  S51 – Ramanan (56)  S52 – Reddy (55)  S53 – Roodenrijs (11)  S54 – Stoll (54)  S55 – Swart (53)  S56 – Takakubo (52)  S57 – Teng (51)  S58 – Vallet (50)  S59 – van Laar (49)  S60 – van Oosterhout (48)  S61 – Verburg (47)  S62 – Wakabayashi (46)  S63 – Wolfe (103)  S64 – Woolfrey (45)  S65 – Wright (44) | Persistency of symptoms and disease activity (PSD)  PSD1 – ACR  PSD2 – DAS  PSD3 – Disease duration  PSD4 – ESR or CRP  PSD5 – Failure to achieve disease remission  PSD6 – Fatigue  PSD7 – Functional Score  PSD8 – HAQ or CHAQ  PSD9 – Imaging or radiographic damage  PSD10 – Joint Count  PSD11 – Joint damage or replacement  PSD12 – New joint activity  PSD13 – Pain  PSD14 – Patient Global  PSD15 – Persistency of symptoms and disease activity  PSD16 – Physician determined or GA  PSD17 – Presence or absence of inflammation  PSD18 – RAPID3  PSD19 – SDAI or CDAI  PSD20 – Severe, erosive or progressive terms used  PSD21 – Stiffness  PSD22 – SUN  Resistance to multiple drugs with different mechanisms of action (RMD)  RMD1 – Despite, previously, failed, unresponsive terms used  RMD2 – Drug discontinuation  RMD3 – Drug duration specified  RMD4 – Drug intolerance or toxicity  RMD5 – Drug regime specified  RMD6 – Failed drugs named  RMD7 – Resistance to multiple drugs  RMD8 – Specified number or Class of drugs failed  RMD9 – Steroid use or dependency  RMD10 – Switching drugs  Other contributing factors (OCF)  OCF1 – Adverse event  OCF2 – Anti-drug Antibodies  OCF3 – Biomechanical or degenerative drivers  OCF4 – Checking diagnosis  OCF5 – Checking relevant treatment given  OCF6 – Co-morbidities or extra-articular manifestations  OCF7 – Other contributing factors  OCF8 – Serology RF or Anti-CCP  OCF9 – Treatment as outpatient |
