# Supplementary material for: Summarizing current refractory disease definitions in rheumatoid arthritis and polyarticular juvenile idiopathic arthritis: systematic review
Source: Rheumatology (Oxford). 2021 Mar 12;60(8):3540–52. doi: 10.1093/rheumatology/keab237 (PMC8328502; doi:10.1093/rheumatology/keab237)
Supplement: keab237_Supplementary_Data [file keab237_supplementary_data.zip › rhe-20-2738-File008.docx]

Supplementary Table S7: Characteristics of Refractory Disease Definitions

| Reference | Definition Name | Verbatim Definition | Definition Generation |
| --- | --- | --- | --- |
| Abinun et al., 2016 (101) | Refractory JIA | Patient 4 was a 13-year-old girl whose rheumatoid factor + poly-JIA was diagnosed at 4 years of age. She had progressive and debilitating polyarthritis refractory to conventional (corticosteroids, methotrexate) and biological (infliximab, etanercept, anakinra, rituximab) DMARDSs (JADAS-10 score over the years 24–30). | Not specified |
| Albers et al., 2014 (100) | Unremitting disease course | Inactive disease was defined as the absence of clinically active arthritis, the lowest possible physician’s global assessment and an ESR <20 mm/h (following a modified definition of clinically inactive disease). The cumulative time spent in active disease in the first 2 years was determined and formed the basis for the categories of disease course: remitting (≤35% active disease), intermediate (35–65% active disease) and unremitting (≥65% active disease). Steroids, csDMARDs and ETN were treatments used. | Not specified |
| Al-Herz et al., 2017 (99) | RA Patients Resistant to Biologic Therapy | Patients were divided into two main groups, biologic therapy resistant defined as patients previously switched from a biologic agent or who are currently not responding to biologic therapy (DAS28 > 3.2). Biologic agents were then divided into anti-TNF and non-anti-TNF agents. In addition, patients who were resistant to >3 biologics were further studied. | Not specified |
| Alvaro-Gracia et al., 2017 (98) | Refractory RA | Eligible patients were adults with a diagnosis of RA for ≥6 months, treated with at least one non-biological agent and with previous failure (inefficacy according to investigator judgement) to at least two biologics (any of the European League Against Rheumatism (EULAR) approved biologics for RA). Patients’ EULAR Disease Activity Score (DAS28— erythrocyte sedimentation rate (ESR)) had to be >3.2; they had to have four tender joints to palpation and four swollen joints (based on a 68/66-joint count) and had to be receiving treatment on an outpatient basis. | Not specified |

| Reference | Definition Name | Verbatim Definition | Definition Generation |
| --- | --- | --- | --- |
| Arjun et al., 2018 (97) | Refractory RA | In this prospective study, 63 patients with active disease, measured by the EULAR DAS28‑ESR ≥3.2, who had failed conventional therapy after at least 3 months of treatment with at least 2 csDMARDs and/or antitumour necrosis factor (anti‑TNF) agents (Infliximab/Etanercept) either standalone, or in combination, were initiated on Abatacept in fixed doses. | Not specified |
| Baxter et al., 2013 (96) | Treatment Resistant RA | Severe active RA (>2 biologic therapies, DAS-28>3.2) | Not specified |
| Becede et al., 2019 (95) | Refractory RA | (i) disease activity: ongoing moderate or high disease activity for at least 12 months determined by SDAI/CDAI for two consecutive clinical visits over the period of at least six months; (ii) treatment failure: insufficient response to at least three courses of DMARDs, including at least one bDMARD; (iii) sufficiently long follow-up time: total time on therapy for at least 18 months. | Statistical analysis using case-control cohort design and sensitivity/validation modelling analysis |
| Beukelman et al., 2011 (94) | Poor Prognostic Factors - Recommendation for switching to 2nd anti-TNF | Following 4 months on first TNFa inhibitor, Moderate (less than three but greater than low disease activity) or High disease activity (at least three of the following: 8 or more active joints, ESR or CRP level greater than twice upper limit of normal, Physician GA ≥7 of 10 or Patient/parent GA ≥5 of 10), irrespective of poor prognostic features (Arthritis of the hip or cervical spine, Positive rheumatoid factor or anti–CCP antibodies or Radiographic damage (erosions or joint space narrowing by radiograph)) | Interdisciplinary panel consensus (International) |
| Blazina et al., 2016 (93) | Refractory Disease | Treatment of Polyarthritis JIA Refractory Disease is with anti-IL-6 (TOC) or ABT after failure of 3 lines of treatment comprising of first line MTX, and second and third line anti-TNF. | Not specified |
| Boers et al., 2015 (92) | Unmitigated failure | Patients experiencing no ACR20 response but at least one adverse event (non-serious or serious) at 6 months (ETN) | Outcome Measures in Rheumatology (OMERACT) Initiative |
|  |  |  |  |
| Reference | Definition Name | Verbatim Definition | Definition Generation |
| Bou et al., 2015 (91) | Refractory Uveitis | Persistent inflammatory activity despite topical corticosteroids, cycloplegics, methotrexate, oral steroids, subtenon injection, adalimumab, and infliximab. | Interdisciplinary panel consensus (National) |
| Breban et al., 1999 (90) | Refractory RA | Their mean age was 48 years (range 32–62 years). The mean duration of RA was 7 years (range 1.5–16 years). All were seropositive for rheumatoid factor (RF), had erosive arthropathy, and were in Steinbrocker functional class III or IV. All patients had severe RA at the time of ID-CYC administration, with a DAS of .3.7 All patients had previously been treated unsuccessfully with each of the following DMARDs: hydroxychloroquine (HCQ), intramuscular gold salts, methotrexate, sulfasalazine, D-penicillamine or tiopronin, and CSA. Each had received multiple courses of corticosteroids that were insufficiently effective. The fourth patient had received anti-CD4 monoclonal antibody (mAb) therapy, all without clinical effect. | Not specified |
| Brown 2015 (89) | Refractory RA | This retrospective case series highlights the clinical features and management of five patients with refractory RA who received ACTH gel based on its proposed mechanism of action after failing several previous therapies (defined as failure to achieve disease remission with a previous treatment). Case 1: Previously failed treatment with adalimumab, etanercept, abatacept, rituximab, tofacitinib, infliximab and prednisone (not a candidate for tocilizumab because of a history of diverticulitis) with RAPID-3=18.7, PainVAS=9.5 and Fatigue=7, Case 2: Previously failed treatment with Etanercept, adalimumab, tocilizumab, abatacept, rituximab, prednisone (discontinued methotrexate due to hair loss) with RAPID-3=20.8, PainVAS=7.5 and Fatigue=7, Case 3: Previously failed treatment with Adalimumab, certolizumab, abatacept, tocilizumab, prednisone with RAPID-3=20.2, PainVAS=8 and Fatigue=5.5, Case 4: Previously failed treatment with Adalimumab, etanercept, tofacitinib (not a candidate for tocilizumab due to history of diverticulitis) with RAPID-3=8, PainVAS=2.5 and Fatigue=2.5, Case 5: Previously failed treatment with Etanercept, adalimumab, abatacept, tocilizumab, prednisone (unable to use methotrexate due to liver function test abnormalities) with RAPID-3=18.8, PainVAS=9 and Fatigue=9. | Not specified |

| Reference | Definition Name | Verbatim Definition | Definition Generation |
| --- | --- | --- | --- |
| Brulhart et al., 2006 (88) | Refractory to anti-TNF | Ten patients fulfilling the 1987 American College of Rheumatology diagnostic criteria for rheumatoid arthritis and refractory to at least one anti-TNF agent for >3 months were prospectively enrolled to start RTX. All of them had active disease (median DAS28 5.28, IQR: 4.6–6.3) | Not specified |
| Buch 2018 (16) | Refractory RA | a) resistance to multiple therapeutic drugs with different structures and mechanisms of action, b) failure of at least one anticytokine (TNF and/or IL-6 directed) and one cell-targeted (B cell depletion and/or T cell costimulation blockade) bDMARD. Intrinsic refractory: persistent inflammation, no ADA (with/without secondary damage). Pharmacokinetic refractory: persistent inflammation with ADA. False refractory: absence of inflammation; other (biomechanical±degenerative) drivers. | Clinical Opinion/Experience |
| Carubbi et al., 2016 (87) | Refractory Monoarthritis | Clinical evidence of a persistent arthritis or a new single joint arthritis (shoulder, elbow, wrist, metacarpophalangeal joint, proximal interphalangeal joint, hip, or knee) despite stable doses of an anti-TNFα agent (infliximab, etanercept, adalimumab) in combination with one or more disease modifying anti-rheumatic drugs (DMARDs) for at least 3 months | Not specified |
| de Hair et al., 2018 (12) | Difficult-to-Treat RA | Persistency of signs and/or symptoms suggestive of inflammatory RA disease activity, despite prior treatment with csDMARDs and at least two bDMARDs. Contributing factors: (i) factors increasing the risk of drug ineffectiveness and recurrent adverse drug reactions (immunologic mechanisms, Smoking, Pharmacogenetics, Immunogenicity of bDMARDs), both of which may result in drug discontinuation; (ii) factors, such as comorbidities and negative disease outcomes (secondary FM/OA, Non-Adherence, Inappropriate medication use, joint damage), which hamper proper grading of RA activity and of effectiveness of medication. | Clinical Opinion/Experience |
| Di Poi et al., 2014 (86) | Refractory to several treatments | 76 years old female patient with chronic arthritis anti-CCP and RF positive, though non-erosive, from 2003, refractory to several treatments (failed methotrexate, leflunomide, etanercept, rituximab) | Not specified |

| Reference | Definition Name | Verbatim Definition | Definition Generation |
| --- | --- | --- | --- |
| Eklund & Joensuu 2003 (86) | Active RA refractory to therapies | RA was required to be refractory to prior therapies including MTX at a dose of 10mg or higher per week given with a glucocorticoid, and with at least one other DMARD. Other inclusion criteria included a swollen joint count ≥6, and at least two of the following three findings: 1) the tender joint count ≥6, 2) a serum CRP level ≥15mg/L or the blood ESR ≥28mm/h; or 3) significant morning stiffness lasting at least for 45 minutes. After obtaining informed consent, one male and two female patients with severe erosive seropositive RA fulfilling the revised criteria of ACR were enrolled. Their disease was resistant to DMARDs, they all had failed MTX used either alone or in a combination with other DMARDs, and all had also failed therapy with INF (two due to side effects and one because of lack of efficacy). | Not specified |
| Emery et al., 2015 (102) | Comprehensive Disease Control Non-Achievers | Patients who achieved either none or any one or two (partial achievers) of the following: a) DAS28(CRP)<2.6, b) HAQ-DI<0.5, ≤0.5 unit change from baseline in mTSS (hands/wrist and feet radiographs) | Not specified |
| Farah et al., 2016 (84) | RTX-Refractory RA f | To evaluate the long-term efficacy and safety of TCZ in real patients with RA refractory to synthetic DMARDs, anti-TNF agents and B cell depletion therapy with RTX, beyond 2 years. | Not specified |
| Fernandes et al., 2017 (83) | Refractory | Only 3 of these could be labelled “refractory”, having previously used 3 biologic agents. | Not specified |
| Fitton et al., 2019 (82) | Multi-targeted therapy Refractory RA | To identify those who have been prescribed 2 or more classes of targeted therapies. Grouped results as 2 class bDMARD, 3 or all 4 classes bDMARD, or Multi bDMARD (min. 2 class) + JAKi. | Not specified |
| FitzGerald et al., 2018 (81) | Treatment-Refractory | Pts were treatment-refractory as evidenced by 53% having received ≥2 prior bDMARDs | Not specified |
| Gaylis et al., 2019 (80) | Multi-drug Refractory RA | The MicroRegulater was implanted on the left vagus nerve in 14 patients with active RA and prior insufficient response to ≥2 bDMARDs or JAK inhibitors with ≥2 different modes of action. | Not specified |

| Reference | Definition Name | Verbatim Definition | Definition Generation |
| --- | --- | --- | --- |
| Genovese et al., 2018 (79) | Refractory to bDMARDs | Patients had active rheumatoid arthritis despite treatment for at least 3 months with at least one bDMARD, or had intolerance or toxicity to bDMARDs. Active rheumatoid arthritis was defined as a swollen joint count out of 66 joints (SJC66) of at least six and a tender joint count out of 68 joints (TJC68) of at least six at the screening and baseline visits, with a high sensitivity C-reactive protein (hsCRP) concentration of at least 3 mg/L at screening (upper limit of normal, 2·87 mg/L). | Not specified |
| Gillis et al., 2017 (78) | Refractory RA | Each patient was required to have RA for at least 2 years, an ACR functional status between one and three, and been treated with at least three therapeutic agents with a different mechanisms of action for >3 months and still have active disease, defined by six tender and six swollen joints. Erythrocyte sedimentation rate (ESR) >28 or C-reactive protein (CRP) >1.2 times the normal limit was required; however, waiver of these particular criteria was at the discretion of the investigator. Each patient must have maintained a stable treatment regimen for 4 weeks prior to initiation of RCI therapy. | Not specified |
| Gomez et al., 2019 (77) | Refractory RA | She had been treated in the past with various biologics for refractory RA - adalimumab, golimumab, tocilizumab. At the time of presentation, she had been on rituximab (2x1g infusions six-monthly), methotrexate (20mg weekly) and prednisolone (15mg daily), since August 2016. | Not specified |
| Hashmi et al., 2012 (76) | Refractory RA | We report two cases of RTX therapy for refractory active RA. Case 1: A 64-year-old Caucasian woman with longstanding seropositive RA had failed treatment with methotrexate (MTX) and etanercept. Case 2: A 63-year-old Caucasian woman presented with active seropositive RA of 10 years’ duration. She had previously failed DMARD treatment (MTX, sulfasalazine) and anti-TNF therapy (adalimumab, etanercept, and infliximab). | Not specified |

| Reference | Definition Name | Verbatim Definition | | Definition Generation |
| --- | --- | --- | --- | --- |
| Hayes et al., 2014 (75) | Refractory RA | A 62 year old male with seronegative RA previously treated with MTX, represented 10 years after his initial diagnosis. His disease proved refractory even with reintroduction of MTX; HCQ and SSZ were inefficacious or had side effects. He was then treated with adalimumab, and when he failed to respond, with rituximab, remaining on MTX throughout. Six months after rituximab (to which he had failed to respond) the patient developed progressive dyspnoea and dry cough, prompting a CT chest which revealed ground glass changes in lower lobes consistent with early interstitial pneumonitis. | | Not specified |
| Heaf et al., 2018 (74) | Biologic Refractory Disease | Patients were classified as refractory on the date they commenced their third biologic. | | Not specified |
| Isaacs 2019 (73) | Refractory RA | My approach to the patient with refractory RA revolves around three questions: 1. Does the patient definitely have RA? 2. Is the disease definitely active? 3. Is the patient truly refractory? In terms of the first question, this is generally straightforward if the patient is seropositive for ACPA but, of course, diseases can co-exist. If they are seronegative (which is common in my refractory clinic), it is important to confirm the diagnosis by rehearsing the history of the disease as well as other relevant history (family history etc.). The second question is probably the commonest cause for debate, largely because of the nuances of the DAS28. Patients in my refractory clinic not uncommonly have a DAS28 that is dominated by patient VAS and tender joints. This, of course, does not exclude inflammatory disease but accessory investigations, particularly musculoskeletal ultrasound, can be very useful under these circumstances. And, thirdly, it is again important to obtain a robust treatment history. This is not always straightforward with a tertiary or quaternary referral but is essential information. Has the patient received good trials of all relevant medications, including conventional synthetic DMARDs? In each case was treatment failure due to inefficacy or adverse events? If the latter, were they ‘credible’ adverse events, well documented and in line with the Summary of Product Characteristics? If the patient suffers recurrent infections is this in part attributable to chronic glucocorticoid therapy? If the response to each of the three questions above is ‘yes’, then the patient probably does have refractory RA. | | Clinical Opinion/Experience |
| Reference | Definition Name | | Verbatim Definition | Definition Generation |
| Jois et al., 2007 (72) | Resistant RA | | Patients with RA who were resistant to more than two anti-TNF agents (treated with each agent at least for 3 months) and had persistent disease activity were considered for treatment with rituximab. Persistent disease activity was defined as disease activity score (DAS-28) >5.1 despite anti-TNF therapy. | Not specified |
| Katsicas et al., 2009 (71) | JIA refractory to ETN and/or INF | | All children with a diagnosis of JIA who were nonresponders to MTX (persistence of active polyarthritis despite the use of higher-dose MTX [≥20 mg/m2/week] for at least 3 months), and anti-TNF agents etanercept (0.4– 1 mg/kg of body weight, subcutaneously twice weekly for at least 6 months) and/or infliximab (3–10 mg/kg of body weight), intravenously, at 0, 2 and 6 weeks followed by infusions every 4 weeks thereafter, concomitantly with MTX 7.5–10 mg/week, were included. All patients needed to have active polyarthritis to enter the study. | Not specified |
| Kawashiri et al., 2010 (70) | Refractory to anti-TNF | | Six RA patients, who met the 1987 criteria of the ACR for RA and showed insufficient response to one or two TNF antagonists (infliximab, etanercept, adalimumab) were consecutively enrolled in the study. All 6 patients were considered to be secondary nonresponders to previous TNF antagonists, as decided by each physician. | Not specified |
| Kearsley-Fleet et al., 2018 (9) | Biologic Refractory Disease | | Patients who had been exposed to at least three different classes of bDMARD (irrespective of reason for failure to prior bDMARD) were classified as ‘bDMARD refractory’. | Not specified |
| Klimiuk et al., 2015 (69) | Refractory to anti-TNF | | Patients recruited to the study had active RA and had an inadequate response to 6 months anti-tumour necrosis factor therapies with infliximab (seven patients) and etanercept (five patients). | Not specified |

| Reference | Definition Name | Verbatim Definition | Definition Generation |
| --- | --- | --- | --- |
| Koumakis et al., 2009 (68) | Refractory Arthritis | We describe 2 patients with very severe refractory RA who failed to respond to anti-TNF (ETN, ADA) or rituximab used alone, but who strikingly responded to the combination of rituximab and anti-TNF (ETN) with sustained remission and good tolerance. A 45-year-old woman was diagnosed with RA in 2003, with symmetric polyarthritis of the wrists, with antibodies positive for RF and CCP and increased CRP (68 mg/l). Initial radiographs were normal. A 32-year-old man was diagnosed with RA in 1999, revealed by symmetric swelling of fingers, wrists, and knees. RF and anti-CCP antibodies were negative. Hand radiographs revealed small erosions of carpal bones. | Not specified |
| Kuek et al., 2006 (67) | Refractory Polyarticular JIA | A 26-yr-old woman was first diagnosed with JIA at the age of 8 yrs. Laboratory findings at presentation revealed anti-nuclear antibody positivity but absence of RF. Numerous DMARDs were initiated in an attempt to achieve symptomatic control including methotrexate, sulphasalazine, myocrisin, minocycline and ciclosporin. All of these, however, were discontinued owing to intolerable side effects or poor efficacy. Frequent flares of arthritis were controlled with high-dose corticosteroid treatment; however, the patient developed Cushing’s syndrome and osteoporotic fractures. Her course was further complicated by the need for bilateral hip replacements at the age of 21 yrs. In 1999, the TNF-antagonist etanercept was commenced, which led to a vast improvement in the signs and symptoms of disease and quality of life for 4 yrs. The development of uveitis and worsening arthritis in 2003 prompted a change to infliximab therapy. This second anti-TNF-agent induced rapid remission of both the joint and eye manifestations of disease, although its efficacy waned after 2 yrs. It was eventually terminated after the development of multi-drug-resistant septic osteomyelitis of the mandible following dental extraction. Adalimumab was next employed, and although this third anti-TNF- agent showed an initial biochemical response, there was no clinical benefit. At the time of infusion with RTX, the patient was wheelchair bound with 24 tender and 15 swollen joints and a DAS28 of 8.32. | Not specified |

| Reference | Definition Name | Verbatim Definition | | Definition Generation |
| --- | --- | --- | --- | --- |
| Liang et al., 2012 (66) | Refractory RA | Patients with established RA were steroid dependent or had failed therapies with methotrexate, hydroxychloroquine, leflunomide, sulfasalazine, and at least one TNF-α blocker. All had high disease severity at baseline (DAS28 6.3-7.99). Clinical response to Mesenchymal stem cells transplant was assessed by the EULAR response criteria. | | Not specified |
| Malaviya et al., 2013 (65) | Refractory RA | 45 year old female patient. Severe active refractory seropositive erosive rheumatoid arthritis. Failed treatment with the following conventional DMARDs: 1. Methotrexate 2. Leflunomide 3. Sulphasalazine 4. Myocrisin 5. Cyclosporin 6. Cyclophosphamide 7. Azathioprine 8. Mycophenolate Failed treatment with the following biologic DMARDs: 1. Infliximab 2. Etanercept 3. Adalimumab 4. Rituximab 5. Abatacept 6. Tocilizumab | | Not specified |
| Marchesoni et al., 2005 (64) | Refractory RA to INF and MTX | The inclusion criteria were: a diagnosis of RA (ACR criteria), an age of 18-75 years, no contraindications to the use of CsA, patient’s willingness to participate to the study (written informed consent), and an (original) DAS of ≥ 3.0 despite combined therapy with infliximab (3-5 mg/kg every 6-8 weeks) and MTX (10-15 mg/week) for at least 6 months. | | Not specified |
| Marketos, Bournazos & Ioakimidis, 2018 (63) | Refractory RA | Patient tried and failed Methotrexate, Leflunomide, Etanercept, Golimumab, Infliximab and Tocilizumab, Anakinra and RTX before initiating Canakinumab. | | Not specified |
| Moeller et al., 2018 (62) | Difficult-to-Treat RA | We evaluated ten patients with difficult-to-treat RA DAS28>=4.2 for the presence and severity of Chronic Periodontitis. All patients had active disease and were positive for rheumatoid factor and anti-cyclic citrullinated peptide antibody. Each had an inadequate response to at least two conventional and two biological disease modifying anti-rheumatic drugs. | | Not specified |
| NHS England 2013 (61) | Refractory patients | a) Unacceptably high-dose of corticosteroids on a regular basis to control disease (> 10 mg prednisolone daily), b) Failure to be controlled on 2 or more standard immunosuppressive agents. | | Not specified |
| Reference | Definition Name | | Verbatim Definition | Definition Generation |
| Olofsson et al., 2018 (60) | Unacceptable Refractory Pain | | Unacceptable refractory pain despite inflammation control combining VAS pain >40mm, with CRP <10 mg/L, and ≤1 swollen joint (of 28) at 2 years | Previous work |
| Park et al., 2020 (59) | Treatment non-persistence | | Treatment non-persistence included switching or discontinuing the index bDMARD and was identified through examination of prescription patterns. Discontinuation of the index bDMARD was defined as: (1) having no claim for any bDMARD after the last day of infusion interval since the date of prescription for the index bDMARD; (2) having a gap of >60 days without a claim for the index bDMARD or a different bDMARD, meaning that a claim for any bDMARD after 60 days from the last day of drug supply would be regarded as discontinuation. A switch was defined as a prescription for a bDMARD (not the index bDMARD) within 60 days from the last day of drug supply during the follow-up period. | Not specified |
| Polido-Pereira et al., 2011 (7) | Refractory Disease | | Failure to achieve the predetermined target. The Treat to Target recommendations did not purpose a specific composite measure of disease activity. Applying the most widely used composite measure (DAS28) in this setting would mean that the ideal target to reach is a DAS28 ≤2.6 and, for those who cannot achieve it, the goal would be to keep a DAS28 ≤3.2. | Treat-to-Target Initiative |
| Pontikaki et al., 2013 (58) | Refractory JIA | | To evaluate efficacy and safety of Golimumab in young adults affected by refractory JIA with active polyarthritis (with or without uveitis), non responders to MTX, antiCD20 and anti-IL-1 and intolerant to the first generation of antiTNF (Infliximab, Etanercept and Adalimumab). All patients were evaluated according to EULAR criteria (DAS 28). | Not specified |
| Pope et al., 2018 (57) | Highly Refractory Disease | | Patients had received ≥3 bDMARDs, representing patients with highly refractory disease | Not specified |

| Reference | Definition Name | Verbatim Definition | Definition Generation |
| --- | --- | --- | --- |
| Ramanan et al., 2018 (56) | Anti-TNF refractory JIA associated uveitis | Children aged between 2 and less than 18 are eligible for the trial and at screening must have active uveitis defined as “2” readings of cellular infiltrate in anterior chamber of Standardisation of the Uveitis Nomenclature (SUN) criteria grade ≥ 1+ or more during the preceding 6 weeks. Participants must have failed an anti- TNF agent and have been on at least one anti-TNF agent regardless of dose for at least 12 weeks at any time previously and must have been on MTX for at least 12 weeks (stable dose for 4 weeks prior to screening) and failed. | Not specified |
| Reddy et al., 2013 (55) | Anti-TNF and Rituximab Refractory RA | All had an active disease with a mean DAS28-ESR score of 6.1, which was refractory to at least two conventional DMARDs; two anti-TNF agents and rituximab used sequentially. | Not specified |
| Roodenrijs et al., 2018 (11) | Difficult-to-Treat RA | DAS28 >3.2 OR presence of signs suggestive of active disease (50%), fatigue (42%), ≥2 csDMARDs AND ≥2 b/tsDMARDs (48%), Inability to taper glucocorticoids <5/10 mg prednisone daily (89%) plus Interfering comorbidities, extra-articular manifestations, radiographic progression, and US synovitis | International survey conducted among rheumatologists |
| Stoll et al., 2015 (54) | Refractory TMJ Arthritis | Intra-Articular INF therapy was used in children who had refractory TMJ arthritis as evidenced by ongoing inflammation detected by MRI despite therapy with Intra-Articular Corticosteroids, and in most cases, systemic immunosuppressive therapy as well (bDMARDs: ETN or ABT). Scoring of MRI was performed largely as previously described, with 1 minor modification: altering the score range for joint effusions from 0–4 to 0–3. For each variable, 0 was the best possible score (no disease), and an increase in score over time represented worsening arthritis. | Not specified |
| Swart et al., 2019 (53) | Refractory JIA | Subjects eligible for this study needed to meet all of the following: patients (4-18 years of age) diagnosed with JIA according to ILAR-criteria with active arthritis resistant to intra-articular steroids and systemic use of methotrexate previously failing all biologicals registered for their JIA subtype. There were no biologicals yet registered for (extended) oligoarthritis JIA patients; however, the failure of two classes of biologicals was required for these patients. | Not specified |

| Reference | Definition Name | Verbatim Definition | Definition Generation |
| --- | --- | --- | --- |
| Takakubo et al., 2012 (52) | Treatment Resistant RA | We identified treatment resistant RA patients for whom we added on Tacrolimus to the ongoing medication in our two hospitals retrospectively. Treatment resistance of RA was defined as a DAS28-CRP score, which was over 3.2 at addition of TAC for more than three months. Eight RA patients were treated using add-on TAC together with ETN (TAC/BIO group), and forty-one RA patients were treated using add-on TAC together with non-BIO DMARDs (TAC/non- BIO group). Treatment response criteria were analyzed by EULAR response criteria based on the DAS28- CRP. | Not specified |
| Teng et al., 2009 (51) | Refractory RA | The present study compared the efficacy and safety of 2 B cell depleting strategies (RTX fixed vs on demand retreatment) in 48 patients with refractory RA failing DMARD combination therapy and/or at least 1 TNF blocking agent. | Not specified |
| Vallet et al., 2016 (50) | Refractory Uveitis | Uveitis was considered refractory if treatment with at least 1 immunosuppressive and/or immunomodulatory agent had failed, with treatment failure defined as fulfilment of at least 1 of the following criteria at inclusion: 1) active inflammatory chorioretinal and/or inflammatory retinal vascular lesions (determined by fluorescein angiography), and 2) a reduction of visual acuity due to vitreous haze or macular edema (determined by optical coherence tomography). Response to treatment was evaluated according to the Standardization of Uveitis Nomenclature Working Group criteria. Complete response was defined as a decrease in the level of inflammation (e.g., anterior chamber cells, vitreous haze) to grade 0, associated with regression of retinal vasculitis and complete resolution of macular edema and with a corticosteroid dosage of #10 mg/day at 6 months. Partial response was defined as ≥50% improvement in inflammation and/or a significant regression of retinal vasculitis (e.g., notably asymptomatic peripheral retinal vascular leakage) and of macular edema and a >50% reduction from the initial corticosteroid dosage at 6 months. All other conditions were considered to be nonresponse. | Not specified |

| Reference | Definition Name | Verbatim Definition | Definition Generation |
| --- | --- | --- | --- |
| van Laar et al., 2001 (49) | Active, destructive rheumatoid arthritis refractory to antirheumatic therapy | Inclusion criteria 1) Progressive erosive disease, 2) Failure to respond to at least 4 second line drugs, including maximal tolerable dose of MTX, and combination therapy of at least 2 second line drugs (Failure to TNF blocking agents was not a prerequisite, as these agents were not registered at the time of initiation of the study. However, 4 patients had failed TNF blocking agents in clinical trials.), 3) Active disease as defined by: ≥ 6 swollen joints and ≥ 6 tender joints and ≥ 1 h early morning stiffness or ESR > 28 mm/h, 4) Steinbrocker functional score class II-III, 5) Disease duration ≥ 3 years and 6) Age 18–60 years | Not specified |
| van Oosterhout et al., 2005 (48) | Refractory RA | A 44 year old woman was diagnosed with rheumatoid factor positive (RF+), erosive RA at the age of 31. Despite treatment with (combinations of) disease modifying antirheumatic drugs (DMARDs), including methotrexate, sulfasalazine, gold, azathioprine, prednisone, oral cyclophosphamide, etanercept, and infliximab, smouldering disease persisted, leading to progressive joint destruction. The patient underwent multiple orthopaedic operations during the disease course. | Not specified |
| Verburg et al., 2005 (47) | Treatment-refractory RA | Patients had an established diagnosis of RA according to the ACR criteria, consisting of progressively erosive disease with involvement of the large joints. In all cases, the disease was refractory to treatment with DMARDs, including the maximum tolerable dosages of methotrexate and combination therapy. Four patients had also been treated unsuccessfully with anti-TNF. All patients had a DAS >3.7 at baseline, defined as high disease activity, and progressive erosive disease. | Not specified |
| Wakabayashi et al., 2012 (46) | Refractory RA | We describe three female patients with active, refractory rheumatoid arthritis who were administered with a second course of etanercept after eventually becoming refractory to a first course. Disease activity (DAS28-ESR, CRP and PGA) was high in all three patients before initial etanercept therapy, and each of them had clinically responded by 24 weeks. However, the initial clinical effect was lost between 1.5 and 3.5 years thereafter, and tocilizumab was administered, but the effect was lost again between 3 and 18 months later. Two patients did not respond to subsequent treatment with adalimumab and infliximab. | Not specified |

| Reference | Definition Name | Verbatim Definition | Definition Generation |
| --- | --- | --- | --- |
| Wolfe et al., 2009 (103) | Patient-based Treatment Failure (Non-success) | Patient global at a level >1.25 best separates patients who are not very satisfied with their health (failure/non-success) from those very satisfied (success), regardless of the presence of comorbidity. All scores increase with increasing comorbidity, which must be accounted for when assessing individual patients, by adding 0.5 to the VAS score cutpoint for each comorbidity. For example, a patient with 2 comorbid conditions is in a “treatment success” state, accounting for comorbidity, if her global score is ≤ 2.25. Treatments included csDMARDs and ETN, ADA and INF. | Statistical analysis using modelling |
| Woolfrey et al., 2010 (45) | Refractory JIA | Diagnosis of systemic onset or polyarticular JIA according to ACR. Disease duration >1 yr. Active inflammatory disease during previous 6 months, defined as either: Involving ≥8 joints, including 2 critical joints (defined as neck, shoulders, elbows, wrists, hips, knees, and ankles) or Involving ≥5 joints, including 2 critical joints, plus 2 of the following: active systemic features: ESR >1.5 ULN, CHAQ score ≥1, or RF positive disease. Unresponsive or unacceptable toxicity to aggressive therapy, defined as all of the following: Prednisone (≥0.3 mg/kg/day) for ≥4 months, Methotrexate (1.0 mg/kg/wk) for ≥4 months, Soluble TNFR:Fc twice weekly for ≥4 months, Combination methotrexate plus soluble TNFR:Fc for ≥4 months, Two separate trials combining methotrexate with either: cyclosporine A or azathioprine or cyclophosphamide or sulfasalazine or intra-articular steroids. | Not specified |

| Reference | Definition Name | Verbatim Definition | Definition Generation |
| --- | --- | --- | --- |
| Wright et al., 2015 (44) | Refractory to anti-TNF and RTX | Here, we report the case of a patient with severe and erosive RA who was refractory to anti-TNF therapy and rituximab over a 4-year period, but who eventually achieved disease remission with tocilizumab. The female patient presented in June 2004 at the age of 25 years, with a 6-year history of seronegative RA and failure to respond to standard DMARDs. Her initial DAS28 was 6.76. She commenced treatment with etanercept (25mg twice weekly), which continued for 22 months with a short (8 month) gap due to pregnancy. Disease activity remained high (DAS28 6.06) and so oral MTX (7.5 mg/week) was introduced for a 4-month period before therapy was switched in May 2006 to adalimumab, initially at a dose of 40mg every other week, increasing to 40mg weekly. This was then stopped after a total of 10 months due to lack of efficacy. The patient’s DAS28 at this time was 6.83 (CRP 72 mg/l, ESR 81 mm/h). The patient next received one infusion of infliximab (3 mg/ kg) in May 2007 but was unable to tolerate further doses. A cycle of rituximab (1 g per infusion) was administered in September 2007, but her DAS28 remained high at 5.44 (CRP 45 mg/l, ESR 47 mm/h) 4 months after treatment. At this time, no further biologic therapies were available outside clinical trials, and the patient commenced s.c. MTX (20mg weekly) and oral prednisolone (15mg daily). | Not specified |
