# Supplementary material for: Summarizing current refractory disease definitions in rheumatoid arthritis and polyarticular juvenile idiopathic arthritis: systematic review
Source: Rheumatology (Oxford). 2021 Mar 12;60(8):3540–52. doi: 10.1093/rheumatology/keab237 (PMC8328502; doi:10.1093/rheumatology/keab237)
Supplement: keab237_Supplementary_Data [file keab237_supplementary_data.zip › rhe-20-2738-File009.docx]

Supplementary Table S8 – Hawker Quality Assessment Results

| **Reference** | **Abstract and Title** | **Introduction and aims** | **Method and Data** | **Sampling** | **Data Analysis** | **Ethics and Bias** | **Results** | **Transferability or generalisability** | **Implications and Usefulness** | **Overall** | **Score** |  |
| --- | --- | --- | --- | --- | --- | --- | --- | --- | --- | --- | --- | --- |
| Abinun (101) | 3 | 2 | 3 | 2 | 1 | 1 | 2 | 2 | 3 | 19 | Low (C) | **Key**: |
| Albers (100) | 4 | 3 | 2 | 3 | 4 | 2 | 3 | 3 | 3 | 27 | Medium (B) | Good= 4 |
| Al-Herz (99) | 1 | 1 | 2 | 2 | 1 | 1 | 3 | 2 | 2 | 15 | Low (C) | Fair = 3 |
| Alvaro-Gracia (98) | 4 | 3 | 4 | 3 | 3 | 3 | 4 | 3 | 3 | 30 | High (A) | Poor = 2 |
| Arjun (97) | 4 | 3 | 4 | 2 | 3 | 2 | 3 | 2 | 3 | 26 | Medium (B) | Very Poor = 1 |
| Baxter (96) | 1 | 3 | 2 | 2 | 1 | 1 | 4 | 2 | 2 | 18 | Low (C) |  |
| Becede (95) | 4 | 4 | 4 | 2 | 4 | 1 | 4 | 3 | 4 | 30 | High (A) |  |
| Beukelman (94) | 1 | 3 | 4 | 3 | 3 | 1 | 4 | 3 | 4 | 26 | Medium (B) |  |
| Blazina (93) | 3 | 4 | 1 | 2 | 1 | 1 | 4 | 2 | 4 | 22 | Low (C) |  |
| Boers (92) | 1 | 2 | 3 | 1 | 3 | 1 | 3 | 1 | 2 | 17 | Low (C) |  |
| Bou (91) | 2 | 4 | 1 | 1 | 1 | 1 | 4 | 3 | 4 | 21 | Low (C) |  |
| Breban (90) | 4 | 2 | 3 | 2 | 1 | 2 | 4 | 2 | 3 | 23 | Low (C) |  |
| Brown (89) | 3 | 3 | 4 | 3 | 3 | 2 | 4 | 3 | 3 | 28 | Medium (B) |  |
| Brulhart (88) | 1 | 2 | 3 | 2 | 1 | 1 | 3 | 2 | 3 | 18 | Low (C) |  |
| Buch (16) | 4 | 4 | 1 | 2 | 1 | 1 | 4 | 2 | 4 | 23 | Low (C) |  |
| **Reference** | **Abstract and Title** | **Introduction and aims** | **Method and Data** | **Sampling** | **Data Analysis** | **Ethics and Bias** | **Results** | **Transferability or generalisability** | **Implications and Usefulness** | **Overall** | **Score** |  |
| Carubbi (87) | 3 | 4 | 4 | 2 | 4 | 2 | 4 | 2 | 4 | 29 | Medium (B) | **Key**: |
| de Hair (12) | 3 | 3 | 1 | 2 | 1 | 1 | 4 | 2 | 4 | 21 | Low (C) | Good= 4 |
| Di Poi (86) | 1 | 2 | 4 | 3 | 3 | 1 | 4 | 3 | 3 | 24 | Medium (B) | Fair = 3 |
| Eklund (86) | 4 | 2 | 4 | 3 | 1 | 2 | 4 | 3 | 3 | 26 | Medium (B) | Poor = 2 |
| Emery (102) | 4 | 4 | 4 | 3 | 4 | 1 | 4 | 3 | 4 | 31 | High (A) | Very Poor = 1 |
| Farah (84) | 1 | 4 | 2 | 1 | 2 | 1 | 3 | 1 | 2 | 17 | Low (C) |  |
| Fernandes (83) | 1 | 3 | 2 | 2 | 1 | 1 | 3 | 2 | 1 | 16 | Low (C) |  |
| Fitton (82) | 1 | 2 | 2 | 1 | 1 | 1 | 2 | 2 | 2 | 14 | Low (C) |  |
| FitzGerald (81) | 1 | 2 | 3 | 2 | 3 | 1 | 4 | 2 | 2 | 20 | Low (C) |  |
| Gaylis (80) | 1 | 2 | 3 | 1 | 1 | 1 | 2 | 2 | 2 | 15 | Low (C) |  |
| Genovese (79) | 4 | 2 | 4 | 4 | 4 | 3 | 4 | 4 | 3 | 32 | High (A) |  |
| Gillis (78) | 4 | 4 | 4 | 3 | 3 | 2 | 4 | 3 | 4 | 31 | High (A) |  |
| Gomez (77) | 1 | 3 | 1 | 2 | 1 | 1 | 3 | 2 | 3 | 17 | Low (C) |  |
| Hashmi (76) | 1 | 1 | 2 | 2 | 1 | 1 | 3 | 2 | 2 | 15 | Low (C) |  |
| Hayes (75) | 1 | 2 | 1 | 2 | 1 | 1 | 2 | 2 | 2 | 14 | Low (C) |  |
| Heaf (74) | 1 | 3 | 4 | 1 | 2 | 1 | 3 | 2 | 2 | 19 | Low (C) |  |
| Isaacs (73) | 1 | 2 | 2 | 2 | 1 | 1 | 3 | 1 | 1 | 14 | Low (C) |  |
| Jois (72) | 4 | 2 | 3 | 2 | 2 | 1 | 3 | 2 | 3 | 22 | Low (C) |  |
| Katsicas (71) | 4 | 3 | 4 | 2 | 1 | 1 | 3 | 2 | 3 | 23 | Low (C) |  |
| **Reference** | **Abstract and Title** | **Introduction and aims** | **Method and Data** | **Sampling** | **Data Analysis** | **Ethics and Bias** | **Results** | **Transferability or generalisability** | **Implications and Usefulness** | **Overall** | **Score** |  |
| Kawashiri (70) | 4 | 3 | 4 | 3 | 3 | 2 | 4 | 3 | 3 | 29 | Medium (B) | **Key**: |
| Kearsley-Fleet (9) | 4 | 4 | 4 | 4 | 4 | 2 | 4 | 4 | 3 | 33 | High (A) | Good= 4 |
| Klimiuk (69) | 3 | 3 | 4 | 2 | 3 | 1 | 4 | 2 | 3 | 25 | Medium (B) | Fair = 3 |
| Koumakis (68) | 1 | 1 | 3 | 2 | 1 | 1 | 3 | 2 | 2 | 16 | Low (C) | Poor = 2 |
| Kuek (67) | 1 | 2 | 2 | 2 | 1 | 1 | 3 | 2 | 2 | 16 | Low (C) | Very Poor = 1 |
| Liang (66) | 4 | 2 | 3 | 2 | 2 | 2 | 3 | 2 | 3 | 23 | Low (C) |  |
| Malaviya (65) | 1 | 1 | 1 | 2 | 1 | 1 | 1 | 2 | 2 | 12 | Low (C) |  |
| Marchesoni (64) | 1 | 2 | 3 | 3 | 2 | 2 | 3 | 3 | 3 | 22 | Low (C) |  |
| Marketos (63) | 4 | 1 | 3 | 3 | 2 | 1 | 3 | 2 | 3 | 22 | Low (C) |  |
| Moeller (62) | 1 | 2 | 4 | 2 | 1 | 1 | 3 | 2 | 2 | 18 | Low (C) |  |
| NHS England (61) | 1 | 1 | 1 | 1 | 1 | 1 | 4 | 2 | 4 | 16 | Low (C) |  |
| Olofsson (60) | 1 | 3 | 3 | 1 | 2 | 1 | 2 | 2 | 2 | 17 | Low (C) |  |
| Park (59) | 4 | 3 | 3 | 2 | 3 | 2 | 4 | 2 | 4 | 27 | Medium (B) |  |
| Polido-Pereira (7) | 2 | 4 | 1 | 1 | 1 | 1 | 4 | 2 | 4 | 20 | Low (C) |  |
| Pontikaki (58) | 1 | 2 | 2 | 2 | 1 | 1 | 2 | 2 | 2 | 15 | Low (C) |  |
| Pope (57) | 1 | 2 | 3 | 1 | 1 | 1 | 3 | 1 | 2 | 15 | Low (C) |  |
| Ramanan (56) | 4 | 4 | 4 | 4 | 4 | 4 | 1 | 3 | 2 | 30 | High (A) |  |
| Reddy (55) | 1 | 2 | 3 | 2 | 2 | 1 | 3 | 2 | 2 | 18 | Low (C) |  |
| Roodenrijs (11) | 4 | 3 | 4 | 2 | 3 | 1 | 4 | 2 | 4 | 27 | Medium (B) |  |
| Stoll (54) | 3 | 2 | 4 | 2 | 3 | 2 | 4 | 2 | 2 | 24 | Medium (B) |  |
| **Reference** | **Abstract and Title** | **Introduction and aims** | **Method and Data** | **Sampling** | **Data Analysis** | **Ethics and Bias** | **Results** | **Transferability or generalisability** | **Implications and Usefulness** | **Overall** | **Score** |  |
| Swart (53) | 4 | 3 | 4 | 4 | 3 | 3 | 3 | 4 | 2 | 30 | High (A) | **Key**: |
| Takakubo (52) | 4 | 2 | 4 | 3 | 4 | 1 | 4 | 4 | 4 | 30 | High (A) | Good= 4 |
| Teng (51) | 1 | 2 | 3 | 2 | 1 | 1 | 3 | 2 | 3 | 18 | Low (C) | Fair = 3 |
| Vallet (50) | 4 | 3 | 4 | 3 | 4 | 2 | 4 | 3 | 4 | 31 | High (A) | Poor = 2 |
| van Laar (49) | 3 | 2 | 3 | 2 | 3 | 1 | 3 | 2 | 3 | 22 | Low (C) | Very Poor = 1 |
| van Oosterhout (48) | 2 | 1 | 2 | 2 | 1 | 2 | 3 | 2 | 2 | 17 | Low (C) |  |
| Verburg (47) | 4 | 2 | 3 | 2 | 2 | 1 | 3 | 2 | 3 | 22 | Low (C) |  |
| Wakabayashi (46) | 2 | 2 | 3 | 2 | 1 | 1 | 4 | 2 | 3 | 20 | Low (C) |  |
| Wolfe (103) | 4 | 3 | 4 | 2 | 4 | 1 | 4 | 2 | 3 | 27 | Medium (B) |  |
| Woolfrey (45) | 2 | 2 | 2 | 2 | 1 | 1 | 3 | 2 | 2 | 17 | Low (C) |  |
| Wright (44) | 1 | 2 | 3 | 2 | 1 | 2 | 3 | 2 | 2 | 18 | Low (C) |  |

Each of the nine domain is ranked 1 for ‘very poor’ to 4 for ‘good’: 1) abstract and title, 2) introduction and aims, 3) methods and data, 4) sampling, 5) data analysis, 6) ethics and bias, 7) findings/results, 8) transferability/generalizability and 9) implications and usefulness. Studies are scored between 9-36 and deemed high-quality (A) if awarded 30–36 points, medium quality (B) if 24–29 points and low quality (C) if 9–23 points.
